# Image-Guided Enhanced PDT/PTT Combination Therapy Using Brominated Hemicyanine-Loaded Folate Receptor-Targeting Ag_2_S Quantum Dots

**DOI:** 10.1021/acs.bioconjchem.3c00096

**Published:** 2023-04-20

**Authors:** Eda Celikbas, Ayca Saymaz, Hande Gunduz, Irem Koc, Ece Cakir, Alphan Sennaroglu, Safacan Kolemen, Havva Yagci Acar, Kubra Onbasli

**Affiliations:** † Department of Chemistry, Koç University, Rumelifeneri Yolu, Sariyer, Istanbul 34450, Turkey; ‡ Nanofabrication and Nanocharacterization Centre for Scientific and Technological Advanced Research, Koç University, Istanbul 34450, Turkey; § Graduate School of Materials Science and Engineering, Koç University, Rumelifeneri Yolu, Istanbul 34450, Turkey; ∥ KUYTAM, Koç University Surface Science and Technology Center, Koc University, Rumelifeneri Yolu, Istanbul 34450, Turkey; ⊥ Departments of Physics and Electrical-Electronic Engineering, Koç University, Rumelifeneri Yolu, Istanbul 34450, Turkey; # Department of Metallurgical and Materials Engineering, Istanbul Technical University, Istanbul 34469, Turkey

## Abstract

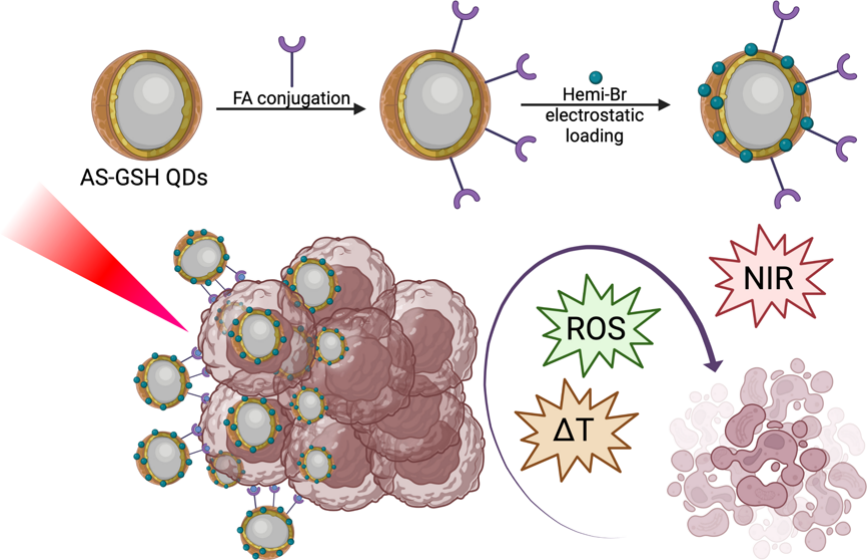

Tumor-targeting nanoparticles and phototherapies are the two major trends in tumor-specific, local cancer therapy with minimal side effects. Organic photosensitizers (PSs) usually offer effective photodynamic therapy (PDT) but require enhanced solubility and tumor-targeting, which may be provided by a nanoparticle. Near-infrared (NIR)-emitting Ag_2_S quantum dots may act as a delivery vehicle for the PS, NIR tracking agent, and as a phototherapy (PTT) agent. A combination of the two provides luminescent dual-phototherapy agents with tumor-specificity and image-guided and enhanced cytotoxicity as a result of synergistic PDT and PTT. In this study, brominated hemicyanine (Hemi-Br), a photosensitizer, was loaded onto folic acid (FA)-tagged, glutathione (GSH)-coated Ag_2_S quantum dots (AS-GSH QDs) to provide enhanced phototoxicity via a photodynamic and mild photothermal effect in folate receptor(+) cancer cell lines at clinically relevant 640 nm irradiation. Final particles (AS-GSH-FA/Hemi-Br) had a hydrodynamic size of 75.5 nm, dual emission at both 705 and 910 nm, and a 93% light-to-heat conversion efficiency under 640 nm laser irradiation. *In vitro* cytotoxicity studies were conducted with folate receptor (FR)-positive HeLa and -negative A549 cell lines to differentiate receptor-mediated uptake. Enhanced phototoxicity on HeLa cells was observed with AS-GSH-FA/Hemi-Br compared to free Hemi-Br and AS-GSH-FA QDs due to increased uptake of the photosensitizer via active targeting and combination therapy, which is especially visible at the safe dose of single agents. Upon irradiation with a 640 nm (300 mW, 0.78 W/cm^2^) laser for 5 min, the viability of the HeLa cells decreased from 64% to 42 and 25% when treated with free Hemi-Br, AS-GSH-FA, and AS-GSH-FA/Hemi-Br, respectively. Overall, AS-GSH-FA/Hemi-Br provides image-guided enhanced PDT/PTT, which may be adopted for different FR(+) tumors.

## 1 Introduction

Developing multifunctional nanoparticles that combine imaging, diagnosis, and therapy became highly desirable in biomedical applications since such materials may reduce the number of agents administered to patients. In recent years, colloidal quantum dots (QDs) have been at the heart of the nanomaterial research in bioimaging, biolabeling, and drug delivery due to their unique properties such as narrow emission bands, tunable size, strong fluorescence, excellent photostability, and a large surface-to-volume ratio.^1,2^ More specifically, near-infrared (NIR)-emitting Ag_2_S quantum dots (AS QDs) appeared as efficient imaging and therapy agents in the current literature.^3−6^ AS QDs are preferred over many existing QDs as they are free of toxic heavy metals and have a low solubility constant (*K*_sp_ = 6.3 × 10^–50^), rendering them highly biocompatible, absorb in the visible/NIR region (700–900 nm), emit in the medical imaging window, and can be synthesized in aqueous media with various functional coatings.^7−9^ Excitation in the visible long wavelengths around 500–550 nm and emission in the NIR region is critical for safety, deep tissue penetration/imaging, and suppressing the tissue autofluorescence, which would lower the effective dose and increase the signal-to-noise ratio. Multifunctional AS QDs have been designed both to visualize the treatment site with their strong NIR fluorescence-providing diagnostics, as well as to deliver the therapeutic cargo efficiently, via a passive enhanced permeability and retention (EPR) effect or receptor-mediated targeting if tagged with a receptor-specific ligand. Hence, AS QDs are very promising theranostic nanoparticles. Several groups produced delivery systems for the clinical chemotherapeutic agents such as doxorubicin (DOX) to visualize the cancer cells utilizing NIR imaging with AS QDs while improving the drug delivery to the tumor, *in vitro* and *in vivo*.^10−13^ Theranostic potential of AS QDs has gone beyond drug delivery, for which most nanoparticles are exploited for, and emerged as a promising photosensitizer for photothermal therapy (PTT) with effective and stable light-to-heat conversion under NIR excitation.^3,4,7,14^ In the clinical application of phototherapy, it is important to visualize where the photosensitizer is located to focus the light source, making PTT a highly local therapy. Therefore, the advantageous NIR emission of AS QDs coupled with long wavelength irradiation to generate local temperature increase renders AS QDs a unique nanoparticle. The current literature exploits the AS-based light therapies either via PTT or in combination with photodynamic therapy (PDT) to provide enhancement over monotreatments.^5,15,16^

Light-based therapies offering locality and a turn-on switch are highly promising for cancer treatment. PTT thermally ablates tumor cells upon the irradiation of photosensitizers (PSs),^14,17^ which usually causes irreversible denaturation of proteins and necrotic cell death.^18^ However, mild hyperthermia may reduce the resistance of the tumor cells to additional therapies, such as chemotherapy or PDT, and enhance the outcome of other therapeutic methods via decreasing the dose and/or irradiation time.^5,7,19−22^

PDT is a clinically approved light-based therapeutic method in which a PS is excited with a specific wavelength to generate reactive oxygen species (ROS) such as singlet oxygen (^1^O_2_) and facilitate a cytotoxic effect to kill tumor cells.^23,24^ However, there are several drawbacks to the clinical applications of PDT, including photobleaching, poor solubility, instability, and rapid clearance of small organic PSs from the bloodstream.^25,26^ Loading PSs to nanoparticles may overcome these problems.^27^ Moreover, by conjugating targeting ligands to the nanoparticle surface, a receptor-mediated, enhanced uptake of the nanoparticles (and PSs) can be facilitated, providing dose and efficiency advantages.^5,7^

PDT has been used in clinical trials for several cancer types in the last few decades, especially on superficial tumors. These include head and neck, esophageal, breast, prostate, and skin cancer in combination with surgery, radiotherapy, and chemotherapy.^28,29^ However, successful attempts at treating superficial tumors have been made using first- and second-generation photosensitizers and red laser or LED irradiation both in *in vivo* models and in clinical trials.^30−35^ Additionally, to overcome the tumor penetration depth limitation, interstitial dosimetry is proposed as a potential alternative in clinical applications.^36,37^

Hemicyanine derivatives are quite attractive in both imaging and phototherapy applications as they offer absorption/emission peaks at the NIR region, water solubility, photostability, and ease of modification toward the development of activity-based agents. It was also well established that the incorporation of heavy atoms (e.g. Br, I) on the core structure improves their ^1^O_2_ generation capacity by enhancing spin–orbit coupling-mediated intersystem crossing. To this end, numerous activatable brominated and iodinated hemicyanine derivatives have been developed so far as highly efficient and cancer-cell-selective PDT agents.^38−42^ Previously, we showed that a brominated hemicyanine core could function as a dual PDT/PTT agent under colaser irradiation operating simultaneously at 640 and 808 nm.^43^ Yet, its water solubility was limited, and it lacked cancer cell targeting. So, cyanine derivatives have been combined with lipid nanoparticles,^44^ polymeric structures,^45,46^ and superparamagnetic iron oxide nanoparticles (SPIONs) to enhance their delivery to tumor cells.^47,48^

In this work, we developed mitochondria-targeting Hemi-Br to load it to folic acid (FA)-tagged NIR-emitting AS QDs for combined PDT/PTT of folate receptor (FR)-positive cancer cells at a single wavelength (640 nm). GSH-coated AS QDs (AS-GSH QDs) were confirmed as successful PTT agents under 808 nm irradiation with a strong intracellular NIR optical signal.^4,7^ This will be the first investigation of AS QD-based PTT at 640 nm with the motivation of achieving PDT/PTT at a single wavelength. As the PDT agent, novel Hemi-Br was synthesized with amine functionality to be loaded to anionic AS QDs. AS-GSH QDs were conjugated with FA via a PEG linker to increase the availability of the targeting moiety. Amine functional Hemi-Br was electrostatically loaded to AS-GSH-FA QDs. FR targeting was investigated on FR-positive HeLa and FR-negative A549 cells. Cellular uptake was monitored with confocal imaging via an NIR camera by facilitating the NIR emission from QDs. The therapeutic effect of the targeted, Hemi-Br-loaded QDs was assessed *in vitro* with and without laser irradiation at 640 nm (300 mW, 0.78 W/cm^2^, 5 min). Light-to-heat conversion efficiency under 640 nm irradiation, the impact of laser irradiation on cell membrane integrity, generation of ROS, and cell death mechanisms were investigated. Such nanoparticles providing tumor-selective, image-guided PDT/PTT combination therapy at a single wavelength of a clinically relevant long wavelength of 640 nm have great potential as novel therapeutic platforms that deserve further attention.

## 2 Results and Discussion

### 2.1 Synthesis and Characterization of AS-GSH-FA/Hemi-Br QDs

The synthesis of Hemi-Br is given in Scheme S1. Initially, compound (1) was synthesized by reacting commercially available 2,3,3-trimethylindolenine with 3-bromopropylamine in acetonitrile. Then, a Knoevenagel reaction was performed between (1) and compound (2) in 1-butanol/benzene to get an IR-780 derivative (3). Prior to the Knoevenagel reaction, (2) was separately synthesized from cyclohexanone through a Vilsmeier–Haack reaction. Later, the free amine groups on (3) were both Boc protected by using Boc anhydride, and the resulting compound (4) was then reacted with 4-bromoresorcinol in the presence of triethylamine (TEA) to obtain the brominated hemicyanine core (5). Finally, standard Boc deprotection in TFA/DCM was performed to obtain Hemi-Br.

**Figure 1:**
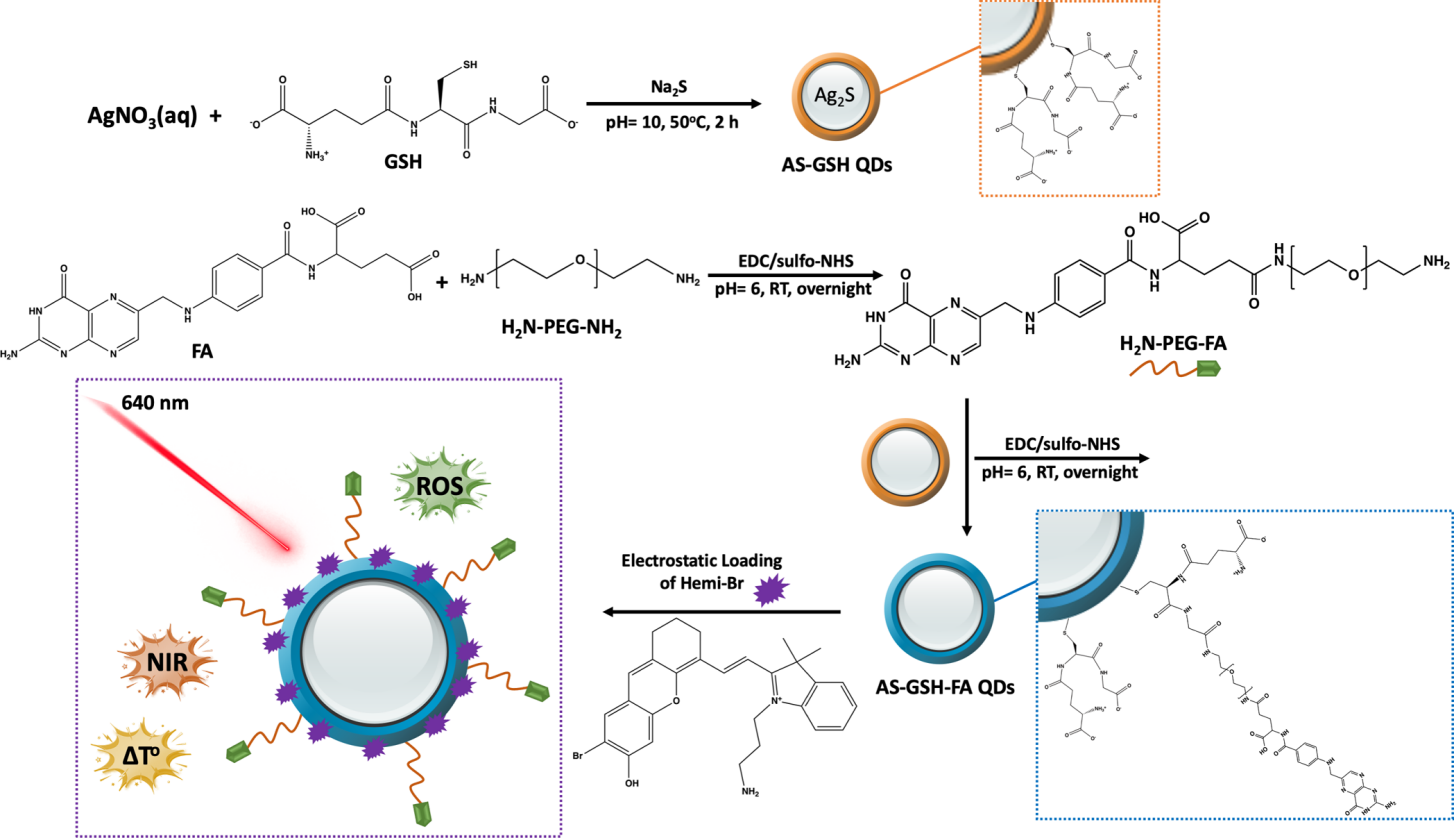
Schematic representation of the synthesis of Hemi-Br-loaded and FA-tagged AS-GSH QDs.

The folic acid-conjugated and Hemi-Br-loaded AS-GSH QDs were prepared, as shown in Figure 1, for image-guided, targeted combined PDT/PTT.

**Figure 2:**
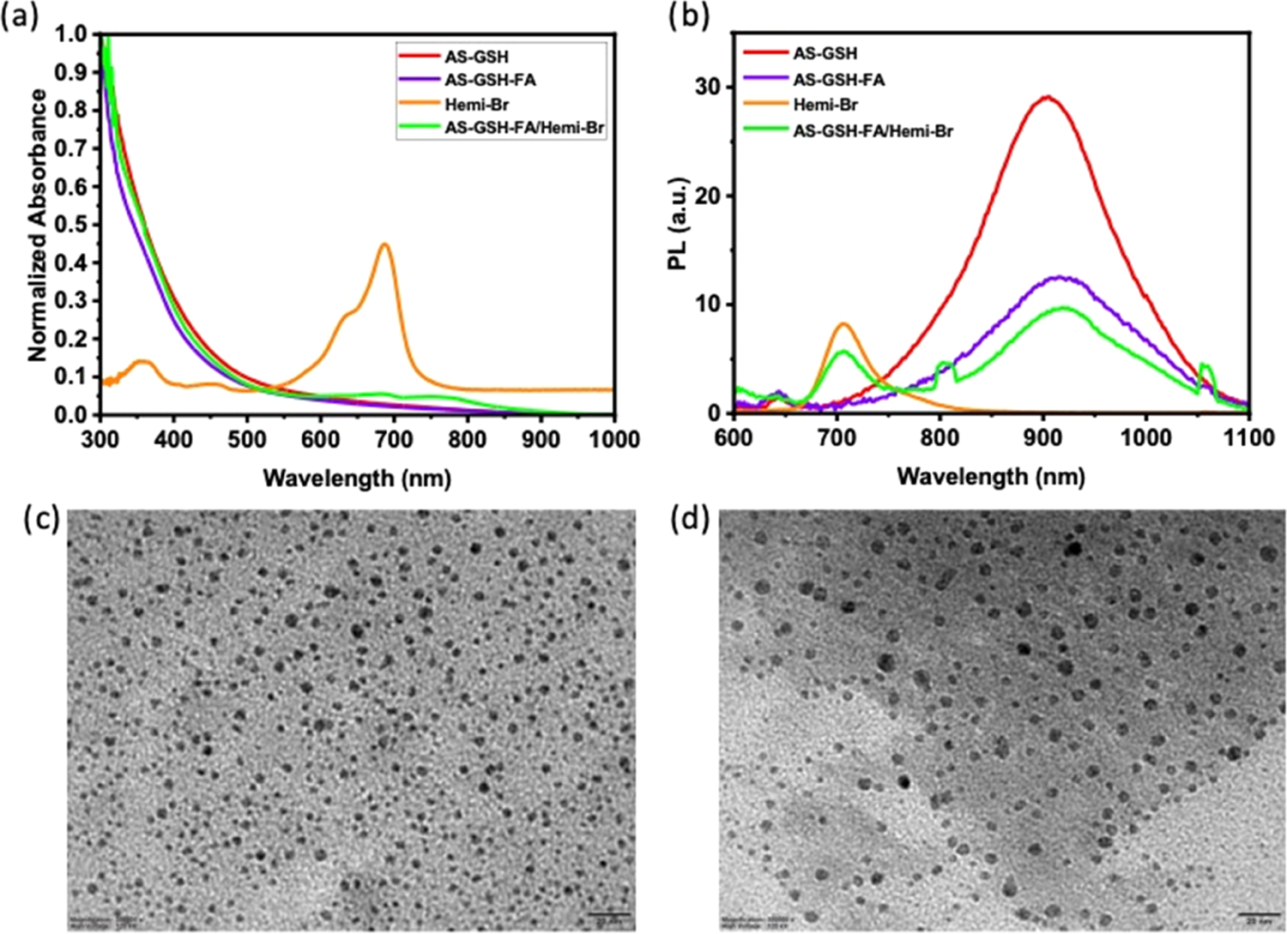
Absorbance (a) and photoluminescence (b) spectra of the QD conjugates and TEM images of AS-GSH-FA (c) and AS-GSH-FA/Hemi-Br QDs (d) (scale bar 20 nm).

Initially, AS-GSH QDs were synthesized in water using GSH as a coating based on our previous work. AS-GSH QDs had an average hydrodynamic size of 2.93 nm and a ζ-potential of −42.2 mV showing strong anionic nature. To conjugate FA to QDs, H_2_N-PEG-NH_2_ was used as a linker to increase the accessibility of FA to the folate receptors on the cell membrane. First, the carboxylic acid groups of FA were activated by EDC/sulfo-NHS in MES buffer and reacted with amine groups of the PEG diamine. Then, the prepared H_2_N-PEG-FA was conjugated to QDs using the same amidation reaction protocol. The FA conjugation efficiency was calculated as 66.33%. The absorbance peak of FA conjugated to AS-GSH-FA QDs was hindered by a strong absorption peak from the QDs on the UV spectrum (Figure 2a). FA conjugation caused a 57% drop in the emission intensity with a 10 nm red shift (Figure 2b), whereas no significant change in hydrodynamic and crystal size was observed, while the ζ-potential was reduced to −34.7 mV (Table 1).

**Table 1:** Hydrodynamic Sizes and ζ-Potentials of Prepared QDs

sample name	PDI	size by number (nm)	ζ-potential (mV)
AS-GSH	0.17	2.94	–42.20
AS-GSH-FA	0.60	5.22	–34.70
AS-GSH-FA/Hemi-Br	0.47	75.50	–21.60
AS-GSH-FA (1 year)	0.16	5.07	–14.90
AS-GSH-FA/Hemi-Br (1 year)	0.55	87.34	–16.70

Additionally, FA conjugation was further confirmed with FTIR spectroscopy (Figure S1). A clearly defined C=O stretching of FA was observed at 1690 and 1655 cm^–1^ for FA and AS-GSH-FA QDs, respectively. The broad characteristic band of hydroxyl groups of glutamic acid in the FA structure was located between 2800 and 3100 cm^–1^ in FA and AS-GSH-FA samples.^49^ Similar peaks at the same range were observed for AS-GSH QDs as they also contain glutamic acid in the structure. The intensity of this peak was reduced in PEG-FA and AS-GSH-FA due to conjugation from the carboxylic acid groups. Additionally, −OH stretching peaks were observed at 3300–3500 cm^–1^. A typical stretching peak of −CH_2_ vibrations was observed for PEG-FA and AS-GSH-FA at 2890 cm^–1^ since FA was conjugated to QDs via a PEG spacer. The strong peak observed for PEG-FA and AS-PEG-FA at 1100 cm^–1^ was associated with the C–O stretching of the ether groups of the PEG. The peaks observed at 847 and 966 cm^–1^ for FA, PEG-FA, and AS-GSH-FA belonged to the C–H bending signal. Overall, the FTIR analysis confirmed the successful conjugation of FA on the surface of the AS-GSH QDs.

As a final step, Hemi-Br was electrostatically loaded to AS-GSH-FA QDs, which was confirmed by ITC (Figure S2). Hemi-Br has an emission peak at 705 nm. After Hemi-Br loading, AS-GSH-FA showed an emission peak of Hemi-Br at 705 nm, with a 22.6% drop in the emission intensity at 916 nm (Figure 2b). Hemi-Br loading increased the hydrodynamic size to 75.5 nm with an expected drop in the ζ-potential. The resulting AS-GSH-FA and AS-GSH-FA/Hemi-Br QDs were homogeneously dispersed and spherical with an average crystal size of 2.5 and 2 nm based on TEM images (Figure 2c,d). Size distribution curves are shown in Figure S3.

Furthermore, the long-term colloidal stability of the designed QDs was investigated over a period of 1 year. The stability of AS-GSH QDs was already reported elsewhere.^4^ As shown in Figure S4a,b, no loss of colloidal stability was observed in terms of UV–vis absorption and PL properties. The emission intensity of the AS-GSH-FA and AS-GSH-FA/Hemi-Br QDs increased over time, whereas no change was observed for the emission intensity of free Hemi-Br at 705 nm. This increasing trend might be attributed to surface perturbations and defects formed during conjugation steps, which reduce the nonradiative recombination and contribute to PL intensity.^50^ Furthermore, all formulations had almost the same hydrodynamic size after 1 year, as shown in Table S1. The ζ-potential of AS-GSH-FA and AS-GSH-FA/Hemi-Br QDs decreased from −34.70 to −14.90 mV and −21.60 to −16.70 mV, respectively, which may be caused by minor impurities formed in the solution and adhered on the surface of the material over 1 year due to usage. Overall, the compositions were colloidally stable for a long period of time.

### 2.2 Investigation of PDT/PTT Potential in Solution

#### 2.2.1 Solution Heating

**Figure 3:**
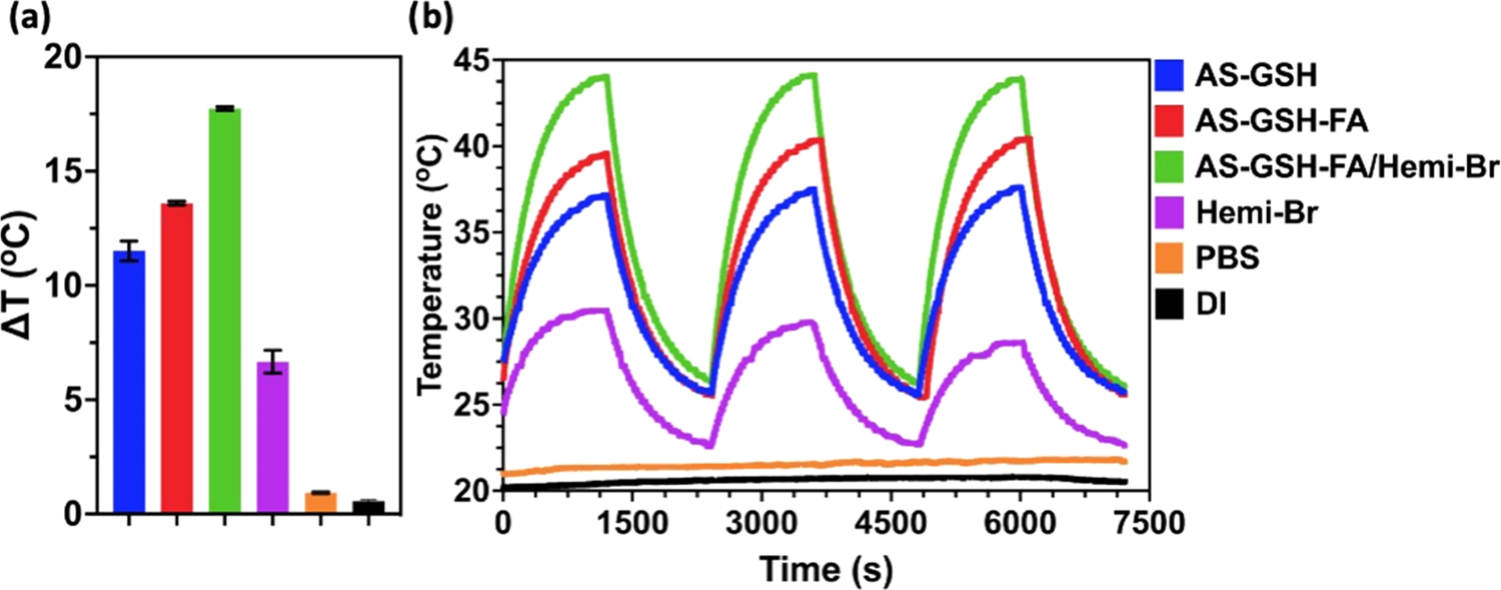
Temperature increase of DI water, PBS, colloidal QDs, and Hemi-Br solution (a) after 20 min of laser irradiation and (b) laser on/off experiments. All experiments were performed at 53 μg Hemi-Br/mL and 300 μg Ag/mL concentrations (*n* = 3). A 640 nm diode laser at 215 mW power was used.

The photothermal heating potential of Hemi-Br, AS-GSH, AS-GSH-FA, and AS-GSH-FA/Hemi-Br was investigated at 300 μg Ag/mL and 53 μg Hemi-Br/mL concentration under 20 min laser irradiation at 640 nm using 215 mW power (Figure 3a). Under these conditions, no temperature increase was detected in the DI water and PBS, indicating that any observed temperature increase is due to Hemi-Br or the QDs. Irradiation of AS-GSH and AS-GSH-FA solutions caused an 11.51 and 13.6 °C increase in solution temperature, respectively, agreeing with strong PTT potential. A moderate temperature increase, 6.67 °C, was observed in the irradiated Hemi-Br solution, suggesting the potential of mild hyperthermia. The maximum temperature increase, 17.74 °C, was observed with AS-GSH-FA/Hemi-Br due to the combined effect of Hemi-Br and QDs. A high light-to-heat conversion efficiency indicates that the photosensitizer can provide sufficient temperature increase even with low incident laser power. This is a highly desired property since it indicates that low levels of laser power may be used for treatment. Light-to-heat conversion efficiencies in water were calculated as 75.55, 87.15, 93.46, and 51.95% for AS-GSH, AS-GSH-FA, AS-GSH-FA/Hemi-Br, and free Hemi-Br, respectively. This is the first report of the light-to-heat conversion efficiency of AS-GSH at 640 nm.

Three cycles of heating/cooling experiments were performed to determine the photostability of Hemi-Br and QDs. The ON/OFF irradiation cycles of AS-GSH, AS-GSH-FA, and AS-GSH-FA/Hemi-Br provided the same Δ*T* after each cycle as expected (Figure 3b). On the other hand, there was an ∼1.2 °C loss after each cycle of the irradiation of free Hemi-Br. These results showed that loading Hemi-Br on the QDs improved its stability and heating efficiency, thus indicating that AS-GSH-FA/Hemi-Br QDs can be used with multiple irradiations as a PTT agent without losing its PTT potential.

Lastly, the temperature increase in the solutions increased with the [Ag] concentration (50–300 μg Ag/mL). As shown in Figure S5, temperature increase was 8.9, 11.4, 14, and 17 °C for AS-GSH-FA/Hemi QDs at [Ag] concentrations of 50, 100, 200, and 300 μg/mL, respectively, after 20 min irradiation with a 215 mW power at 640 nm.

#### 2.2.2 Singlet Oxygen Generation (^1^O_2_) in Solution

^1^O_2_ generation potential of Hemi-Br (10 μM) was investigated initially by using an ^1^O_2_ selective SOSG trap molecule. SOSG emission is quenched by a photoinduced electron transfer (PeT) mechanism,^51^ and the characteristic green emission at 530 nm is restored selectively in the presence of ^1^O_2_. As shown in Figure S6, after the irradiation at 640 nm (215 mW) for 5 min, the emission signal of Hemi-Br significantly increased, indicating photosensitized ^1^O_2_ generation. The singlet oxygen quantum yield of Hemi-Br was found to be 10% in DMSO by using methylene blue as a reference PS^52^ and 1,3-dipehenylbenzofuran (DPBF) as a trap molecule (Figure S6b).

### 2.3 Cytotoxicity

**Figure 4:**
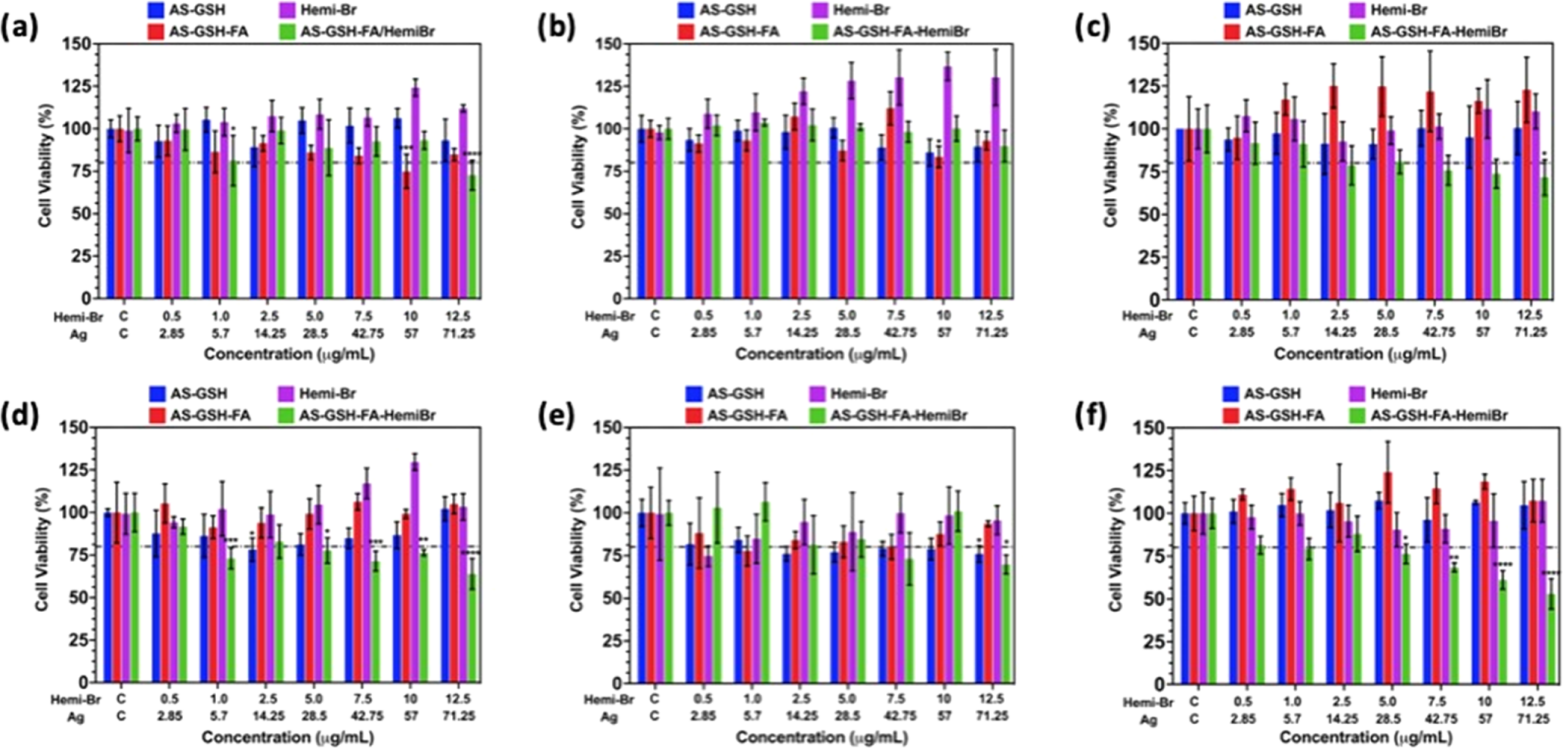
Dose-dependent cytotoxicity results determined by MTT assay for (a) HeLa, (b) A549, and (c) L929 cells after 6 h of incubation. (d) HeLa cells, (e) A549 cells, and (f) L929 cells after 24 h of incubation. The data are expressed as mean ± S.D. (*n* = 5). (0.0332 (*), 0.0021 (**), 0.002 (***), <0.001 (****)).

Dose- and time-dependent cytotoxicities of free Hemi-Br, AS-GSH, AS-GSH-FA, and AS-GSH-FA/Hemi-Br were determined on HeLa (folate-positive cancer), A549 (folate-negative cancer), and L929 (healthy fibroblast) cells using standard MTT assay (Figure 4). Cells were treated with nanoparticles between 2.85 and 71.25 μg Ag/mL or 0.5 and 12.5 μg Hemi-Br/mL concentrations, equivalent to Hemi-Br content of AS-GSH-FA/Hemi-Br, for 6 and 24 h. According to ISO 10993-5, neither the QDs nor free Hemi-Br was toxic to HeLa or A549 cell lines in 6 h of incubation since viabilities were above 80% (Figure 4a,b). These agents did not cause any reduction in viability of L929 cells in 6 h as well (Figure 4c), except AS-GSH-FA/Hemi-Br above 2.5 μg Hemi-Br/mL dose. The cellular viability of L929 cells was decreased to 70–75%.

Similarly, no cytotoxicity was observed when the cells were treated with free Hemi-Br, AS-GSH, or AS-GSH-FA for 24 h. In the case of AS-GSH-FA/Hemi-Br, some dose-dependent cytotoxicity was observed in all three cell lines. The viability of HeLa cells and A549 cells dropped to 63 and 70% at the highest dose (71.25 μg Ag/mL and 12.5 μg Hemi-Br/mL) due to enhanced uptake (Figure 4d,e). The L929 cell line was more sensitive, and its viability was between 75 and 53% at 5.0–12.5 μg Hemi-Br/mL (Figure 4f).

### 2.4 Cellular Uptake

**Figure 5:**
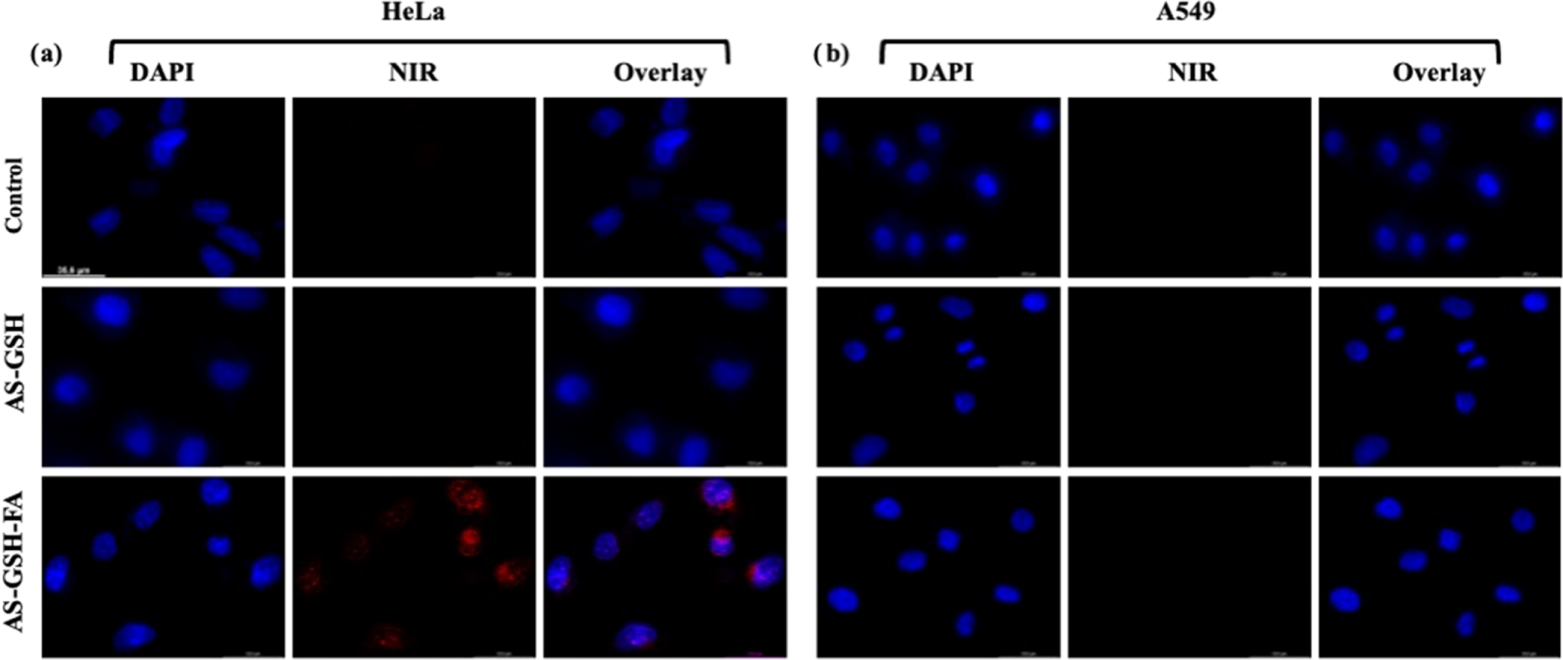
Confocal microscopy images of AS-GSH- and AS-GSH-FA-treated (a) HeLa and (b) A549 cells after 6 h of incubation at 42.5 μg Ag/mL dose. Untreated cells were used as controls. Filters: DAPI (λ_ex_ = 365 nm and λ_em_ = 417–477 nm) and NIR (λ_ex_ = 510–550 nm and λ_em_ = 710 nm long pass filter). The representative scale bar on HeLa (control) = 35.6 μm.

Folate receptor targeting of FA-conjugated QDs was investigated on low (A549) and high (HeLa) FR-overexpressing cells at 42.5 μg Ag/mL after 6 h of incubation (Figure 5). Short incubation time was facilitated to differentiate receptor-mediated endocytosis from the passive uptake of QDs in long incubation. No intracellular NIR fluorescence was observed in HeLa and A549 cells treated with untagged QDs (AS-GSH). A strong optical signal from targeted QDs (AS-GSH-FA) was observed in HeLa cells, whereas no signal was acquired for A549 cells, as expected. Our findings indicate that FA-conjugated QDs can be internalized significantly better by the FR-overexpressing cells at short incubation times, implying effective receptor-mediated endocytosis. Therefore, FA-tagged QDs and Hemi-Br-loaded FA-tagged-QDs can be delivered selectively and efficiently to FR(+) tumors in short incubation and can be detected at the tumor site via NIR optical imaging for image-guided phototherapy.

Intracellular uptake was further quantified by measuring the NIR emission of internalized QDs after 6 h of incubation of the cells with the agents. A specific filter set was used for this experiment at a 528 ± 20 nm excitation and an 818 ± 40 nm emission wavelength. As shown in Figure S7, the fluorescence signal was increased significantly when HeLa cells were incubated with AS-GSH-FA QDs compared to control and AS-GSH QDs. A slight, but not statistically significant, increase in the fluorescence signal was observed from the cells incubated with free Hemi-Br as it has a very low emission signal around 818 nm, as shown in Figure 2. No significant difference was observed between the groups for the A549 cell line, further confirming the efficient receptor-mediated intracellular uptake of FA-tagged QDs. The slightly different uptake levels of free Hemi-Br between the two cell lines could have resulted from the metabolic differences.

### 2.5 *In Vitro* ROS Generation

**Figure 6:**
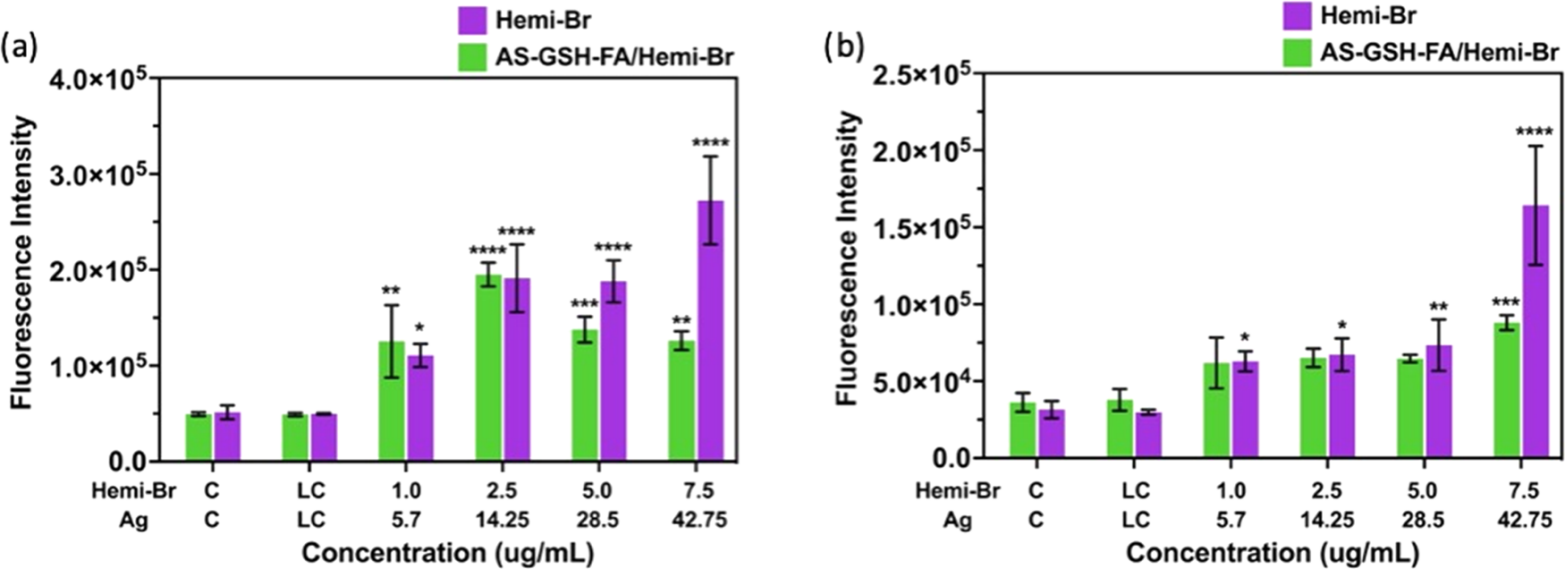
Intracellular ROS levels of (a) HeLa and (b) A549 cells upon incubation with free Hemi-Br and AS-GSH-FA/Hemi-Br after 6 h of incubation and 5 min laser irradiation at 640 nm. The data are expressed as mean ± S.D. (*n* = 3). (0.0332 (*), 0.0021 (**), 0.002 (***), <0.001 (****)).

Reactive oxygen species (ROS) are the major factor of cell death in PDT and a significant contributor to combined PDT/PTT. Therefore, dose-dependent intracellular ROS generation of Hemi-Br and AS-GSH-FA/Hemi-Br was investigated using a cell-permeable ROS sensor, 2′,7′-dichlorofluorescein diacetate (DCFH_2_-DA), which emits in the green region upon oxidation. HeLa and A549 cells were treated with the QDs using the same protocol mentioned in the PDT/PTT combination studies and incubated for 40 min with the dye before laser irradiation. The measurements were performed right after the laser treatment. Both agents provided up to fourfold ROS generation compared to control between 1 and 2.5 μg Hemi-Br/mL (Figure 6a). At higher doses, intracellular ROS amount in the cells treated with AS-GSH-FA/Hemi-Br was lower than those treated with free Hemi-Br. However, this is probably due to the strong absorption of the DCF emission (530 nm) by QDs, which decreases the intensity of the DCF signal coming out from the AS-GSH-FA/Hemi-Br-incubated cells. A similar trend was observed for the A549 cells, as shown in Figure 6b; however, the increase in the ROS levels was only up to 2.5-fold. These results support the phototoxicity difference between the two cell lines (Figure 4).

### 2.6 PDT/PTT Combination Therapy

**Figure 7:**
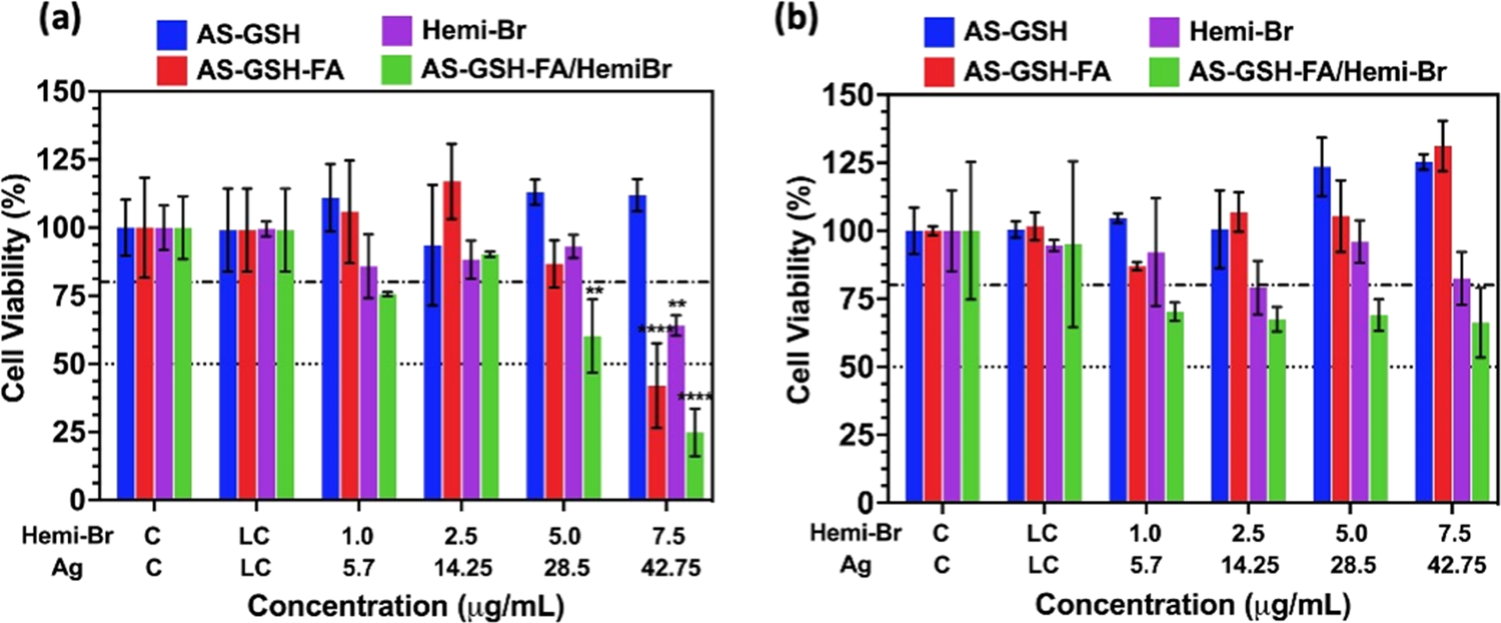
Dose-dependent viability of (a) HeLa and (b) A549 cells treated with free Hemi-Br and QD conjugates for 6 h and irradiated at 640 nm for 5 min. The data are expressed as mean ± S.D. (*n* = 3). (0.0332 (*), 0.0021 (**), 0.002 (***), <0.001 (****)).

*In vitro* PDT/PTT combination potential of QDs was investigated in HeLa and A549 cell lines at a dose range of 1.0–7.5 μg Hemi-Br/mL and 5.7–42.75 μg Ag/mL, which showed no dark toxicity. Cells were incubated with the agents for 6 h and irradiated with a 640 nm laser (300 mW, 0.78 W/cm^2^) for 5 min. No phototoxic effect was observed when the cells were treated with only laser, as shown in Figure 7, as laser control (LC), confirming that the irradiation protocol itself is safe. Although AS-GSH QDs did not cause any phototoxicity in either cell line, strong phototoxicity was observed in HeLa cells treated with AS-GSH-FA QDs, indicating that in the studied range of [Ag] dose, untagged QDs were not internalized in critical amounts to provide PTT; however, FA-tagged ones selectively accumulated in FR(+) HeLa cells via receptor-mediated active targeting (Figure 7a). At 42.75 μg Ag/mL, only 42% of the HeLa cells remained viable, whereas, at the same dose, no phototoxic effect was observed on FR-negative A549 cells (Figure 7b).

Free Hemi-Br induced significant phototoxicity only at the highest studied dose, 7.5 μg Hemi-Br/mL, and more on HeLa cells with 65% viability compared to 80% viability of A549 cells. This suggests that Hemi-Br itself was providing more efficient phototoxicity on HeLa cells, which is in good correlation with the *in vitro* ROS levels (Figure 6) and intracellular uptake quantification (Figure S7).

Phototoxicity was further enhanced when the FR-targeted combination therapy was applied on HeLa cells using AS-GSH-FA/Hemi-Br, while no significant change in the cytotoxicity was observed for A549 cells, which stayed at ∼70% in this dose range. Only 25% of the HeLa cells were viable at 7.5 μg Hemi-Br/mL (corresponding to 42.75 μg Ag/mL). By increasing the concentration of the agent to 12.5 μg Hemi-Br/mL (corresponding to 71.25 μg Ag/mL), the complete death of HeLa cells was observed (Figure S8a). At the same dose, A549 cells showed 50% cell viability, further confirming FR-mediated uptake of the FA-tagged QDs (Figure S8b).

The contribution of the photodynamic effect was investigated by NaN_3_, a ^1^O_2_ quencher, inhibition studies. To this end, HeLa and A549 cells were incubated with either free Hemi-Br/mL or AS-GSH-FA/Hemi-Br (7.5 and 10 μg Hemi-Br/mL corresponding to 42.75 and 57 μg Ag/mL) in the presence or absence of NaN_3_ (5 mM). The cell viability in HeLa cells was remarkably increased, especially at higher doses of Hemi-Br and AS-GSH-FA/Hemi-Br, in the quencher-treated groups, proving the role of ^1^O_2_ in dual-phototherapy action (Figure S9a). In the case of A549 cells, no significant change was detected after NaN_3_ treatment since free Hemi-Br is less effective in A549 and FA-tagged QDs are not selective for this cell line (Figure S9b).

### 2.7 Live/Dead Assay

Live-dead assay using calcein-AM/ethidium bromide staining was used to confirm the drop in viability observed in the MTT assay as cell death. Cells were again treated with the agents for 6 h and then irradiated at 640 nm (300 mW, 0.78 W/cm^2^) for 5 min. As shown in Figure S10, in the absence of laser irradiation, all cells are live indicated by green fluorescence regardless of the cell type in agreement with the MTT assay results (Figure 4). However, after laser irradiation, the red fluorescence of dead cells is clearly visible in HeLa cell lines treated with AS-GSH-FA, AS-GSH-FA/Hemi-Br, and free Hemi-Br. More importantly, a large portion of the HeLa cells was dead when the targeted PTT/PDT combination treatment was applied with AS-GSH-FA/Hemi-Br at 640 nm. Additionally, the morphology of the live cells was also significantly changed. On the other hand, little to no dead A549 cells were observed, further confirming previous results and selective uptake coupled effective killing of the FR(+) cancer cell line.

### 2.8 Mitotracker Assay

**Figure 8:**
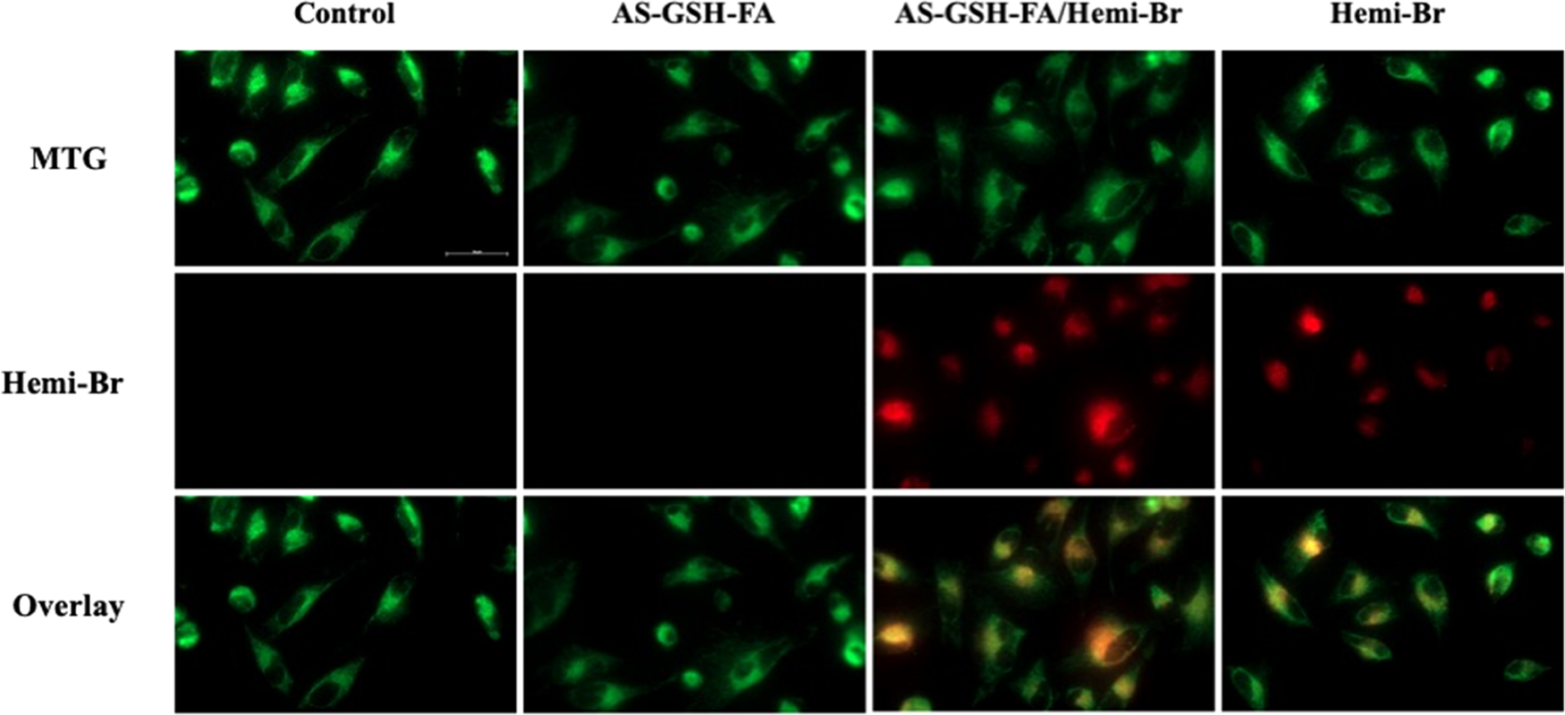
Fluorescence images of the MTG colocalization experiment in HeLa cells. Green: MTG (25 nM, λ_ex_ = 488 nm, λ_em_ = 500–550 nm) and red: Hemi-Br (7.5 μg Hemi-Br/mL, λ_ex_ = 635 nm, λ_em_ = 680–750 nm). The representative scale bar on control = 50 μm.

Mitochondrial colocalization of Hemi-Br was investigated by using a commercial MTG Dye. HeLa cells were treated with AS-GSH, free Hemi-Br, and AS-GSH-FA/Hemi-Br following the same protocol at 7.5 μg Hemi-Br/mL (corresponding to 42.75 μg Ag/mL). After 6 h of incubation, the mitochondria of the cells were dyed with the MTG following the manufacturer’s instructions. As shown in Figure 8, the red fluorescence signal acquired from the Hemi-Br was colocalized with the MTG signal. The calculated Pearson coefficients for the colocalization of MTG with free Hemi and AS-GSH-FA/Hemi-Br were 0.82 and 0.824, respectively (Figure S11). Furthermore, an increased fluorescent signal was observed with AS-GSH-FA/Hemi-Br QDs compared to free Hemi-Br due to enhanced uptake of the nanoparticles by the cells. These results demonstrated the mitochondrial targeting ability of Hemi-Br, indicating a possibility of enhanced therapeutic outcomes.

### 2.9 LDH Release

**Figure 9:**
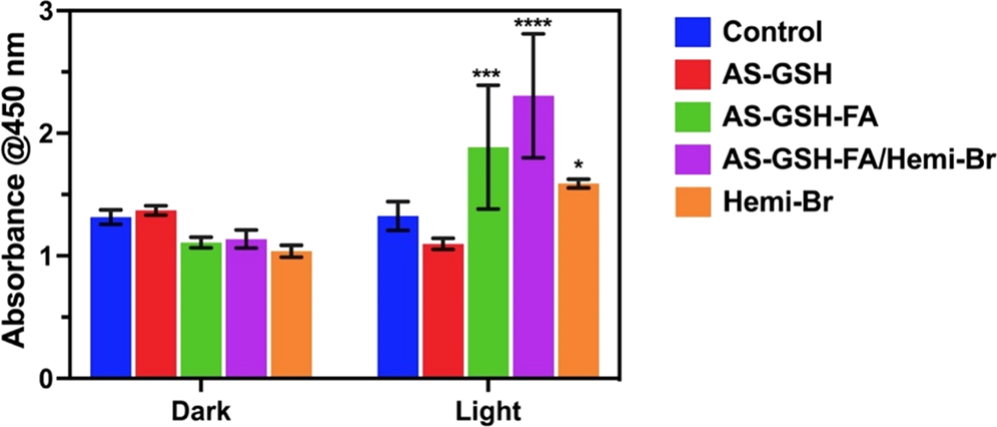
LDH release of HeLa cells exposed to 7.5 μg Hemi-Br/mL (corresponding to 42.75 μg Ag/mL) after 6 h of incubation with and without laser treatment at 640 nm (300 mW, 0.78 W/cm^2^) for 5 min. The data are expressed as mean ± S.D. (*n* = 3). (0.0332 (*), 0.0021 (**), 0.002 (***), <0.001 (****)).

As a key indicator of necrosis and PTT, the LDH enzyme is released when there is damage to the cellular membrane integrity.^53^ LDH levels in HeLa cells were measured with and without laser irradiation after the treatment with free Hemi-Br and QDs at 7.5 μg Hemi-Br/mL (corresponding to 42.75 μg Ag/mL). As expected, no significant increase in the LDH levels was observed without laser irradiation since there was no cytotoxic effect on this concentration in the dark, according to the MTT assay (Figure 7). On the other hand, AS-GSH-FA, AS-GSH-FA/Hemi-Br, and free Hemi-Br showed a significant increase in the LDH levels after 5 min of irradiation at 640 nm (300 mW, 0.78 W/cm^2^) compared to the laser control that was observed with free Hemi-Br and QDs (Figure 9). The highest increase was observed when the cells were treated with AS-GSH-FA/Hemi-Br and underwent irradiation due to targeted and enhanced PTT. These results were entirely in line with the MTT results shown in Figure 7, indicating that the PTT caused necrotic cell death, resulting in LDH release.

## 3 Conclusions

This study achieved a successful synthesis of folate targeting, NIR-luminescent, small AS QDs loaded with Hemi-Br as a novel PDT/PTT dual agent. AS-GSH-FA/Hemi-Br had dual emission peaks, one at 705 nm and the other at 910 nm provided by Hemi-Br and AS-GSH QDs, respectively, which may be exploited for imaging and localization of the agents. AS-GSH-FA/Hemi-Br showed about 93% light-to-heat conversion efficiency, suggesting strong PTT potential at 640 nm. AS QDs are usually utilized for NIR photosensitization, but this first report of their efficiency at 640 nm is critical since, at a single wavelength, both the QD and the Hemi-Br produce phototoxicity. AS-GSH-FA/Hemi-Br efficiently produced ROS and increased LDH levels of HeLa (FR-positive) cells selectively compared to A549 (FR-negative) cell lines due to folate targeting, resulting in higher uptake and stronger phototoxicity. Clearly, AS-GSH-FA both acted as a mild PTT agent and a delivery vehicle for Hemi-Br, improving its phototoxicity. Overall, these results demonstrated an enhanced therapeutical outcome of tumor-targeting-combined PDT/PTT compared to monotherapies.

## 4 Materials and Methods

### 4.1 Materials

All reagents were of analytical grade or of the highest purity. Silver nitrate (AgNO_3_), folic acid (FA), poly(ethylene glycol) diamine (H_2_N-PEG-NH_2_, 2000 MW), and 2-(N-morpholino)ethanesulfonic acid (MES) were purchased from Sigma-Aldrich. Sodium sulfide (Na_2_S) and 1-ethyl-3-(3-dimethylamino propyl) carbodiimide (EDC) were purchased from Alfa Aesar (U.K.). Glutathione (GSH), sodium hydroxide (NaOH), ethanol, acetic acid (CH_3_COOH), and N-hydroxysuccinimide (NHS) were purchased from Merck. Ultracentrifuge tubes with 3 and 10 kDa cutoffs were purchased from Sartorius (Goettingen, Germany).

The Roswell Park Memorial Institute (RPMI) 1640 medium, Gibco Dulbecco’s Modified Eagle Medium (DMEM), l-glutamine, trypsin–EDTA, and penicillin–streptomycin solutions were purchased from Capricorn Scientific GmbH (Germany). Fetal bovine serum (FBS) was obtained from PAN-Biotech (Germany). Thiazolyl blue tetrazolium bromide (MTT) was provided from Biomatik Corp. (Canada). Phosphate-buffered saline (PBS) tablets were purchased from Research Products International (RPI) Corp. A 4% paraformaldehyde solution in PBS was obtained from Santa Cruz Biotechnology, Inc. Further, 96-well plates and 30 mm glass-bottom Petri dishes were purchased from Nest Biotechnology Co. Ltd. (China). HeLa human cervical cancer cells and A549 adenocarcinoma human alveolar basal epithelial cells were provided by the Gozuacik Lab (Koç University, Istanbul, Turkey). Healthy fibroblast L929 cells were gifted from Engin Ulukaya Lab (Istinye University, Istanbul, Turkey). The live/dead cell viability assay was purchased from Thermo Fisher Scientific. LDH assay was purchased from Promega.

All reagents used in the hemicyanine synthesis were commercially available and used without further purification unless otherwise noted. All dry solvents used in reactions were obtained by using standard procedures and dry tetrahydrofuran (THF) was obtained from the Inert PureSolv solvent drying system. Reactions performed under the inert atmosphere were performed in the Schlenk line under argon. Thin-layer chromatography (TLC, Merck Silica Gel 60 F254) was performed by using commercially prepared 0.25 mm silica gel plates and the compounds were visualized under UV light. Column chromatography was performed using thick-walled glass columns and silica Gel 60 (Merck 230–400 mesh). The relative proportions of solvents in chromatography solvent mixtures refer to the volume/volume ratio. ^1^H NMR and ^13^C NMR analyses were recorded by a 500 MHz Bruker Ascend magnet equipped with an Avance NEO console spectrometer using CDCl_3_, MeOD, or *d*_6_-DMSO as the solvents. The chemical shifts are reported in parts per million (ppm) downfield from an internal trimethylsilane (TMS) reference. Coupling constants (*J*) are reported in hertz (Hz), and the spin multiplicities were specified by the following symbols: s (singlet), d (doublet), t (triplet), and m (multiplet). NMR spectra were processed with the MestReNova program. Mass spectra were recorded on a Waters Vion High-Definition mass spectrometer. UV–vis spectra were acquired on a Shimadzu UV1900i spectrophotometer. Fluorescence measurements were done by using an Agilent Cary Eclipse spectrophotometer.

### 4.2 Synthesis of AS-GSH QDs

AS-GSH QD was synthesized as reported in the literature in our previous work. Briefly, AgNO_3_ and GSH were dissolved in 75 mL of deoxygenated water with a molar ratio of Ag/GSH of 1:2 in a round-bottom flask under continuous argon flow. The solution was heated up to 50 °C, and the pH was adjusted to 10 with 1 M NaOH and CH_3_COOH. Na_2_S was dissolved in 25 mL of deoxygenated water, sonicated for 2 min, and then added to the reaction mixture dropwise. The QDs were formed after a 2 h reaction at 50 °C under vigorous mechanical stirring at 500 rpm. QDs were washed with ultracentrifuge filters with a 3 kDa cutoff and stored at 4 °C and in the dark for further use.

### 4.3 Conjugation of AS-GSH QDs with FA

First, FA was conjugated to diamine-PEG (MW 2000 Da) via standard amidation reaction using EDC/sulfo-NHS chemistry in water (pH = 6.0). FA was dissolved in deionized water and the carboxylic acid groups were activated with 5.49 μmol EDC and sulfo-NHS for 30 min at ambient conditions. Then, H_2_N-PEG-NH_2_ (5.49 μmol) was added to the reaction mixture to be conjugated with FA and reacted at room temperature overnight in the dark. The resulting H_2_N-PEG-FA conjugate was washed with deionized water using a 3 kDa ultracentrifuge tube and stored at −20 °C after lyophilization.

The carboxylic acid groups of AS-GSH QDs were activated via the same protocol using EDC/sulfo-NHS chemistry as described above. First, QDs were transferred from water to the MES buffer (0.1 M, pH = 6.0) using ultracentrifuge tubes, and 0.36 mmol of EDC and sulfo-NHS were added to the QD solution and mixed at room temperature for 30 min at 750 rpm. Then, the excess EDC and sulfo-NHS were washed in 3 kDa ultracentrifuge tubes and the buffer solution was changed into PBS (pH = 7.0–7.5). Overall, 15 mg of a H_2_N-PEG-FA conjugate, which correlates to 5 mol % of the surface COOH, was then added to the QDs and reacted at room temperature overnight at 750 rpm. Finally, the resulting conjugates were washed with PBS (3 kDa cutoff) and the amount of FA conjugated to the QD surface was quantified by measuring the absorbance of PEG-FA in the washed solution at 320 nm, using a concentration-dependent calibration curve for PEG-FA.

### 4.4 Synthesis of Hemi-Br

Compounds 1, 2, 3, and 4 were synthesized according to the previously reported methods.^54,55^

#### 4.4.1 Synthesis of (5)

In a round-bottom flask, under an argon atmosphere, 4-bromoresorcinol (0.89 g, 4.70 mmol) was dissolved in DMF (5 mL) and TEA (0.76 mL, 5.49 mmol) was added, and the mixture was stirred at room temperature for 15 min. To this solution, a solution of compound 4 (0.60 g, 0.78 mmol) in DMF (5 mL) was added dropwise via a syringe, and the reaction mixture was heated to 60 °C for 5 h. After the completion, the reaction was cooled to room temperature and the crude mixture was diluted with DCM and then washed with water (3 × 30 mL). Combined organic phases were then washed with brine (30 mL) and dried over Na_2_SO_4_, the solvent evaporated under reduced pressure, and the crude mixture was purified by column chromatography (MeOH/DCM, 5:95) to obtain compound 5 as a dark green solid (0.23 g, 49% yield). ^1^H NMR (500 MHz, CDCl_3_) δ 8.06 (d, *J* = 13.2 Hz, 1H), 7.66 (s, 1H), 7.32–7.27 (m, 3H), 7.06 (t, *J* = 7.4 Hz, 1H), 6.81 (d, *J* = 7.8 Hz, 1H), 6.65 (s, 1H), 5.62 (d, *J* = 13.2 Hz, 1H), 4.67 (br. s, 1H), 3.83 (br. s, 2H), 3.25 (br. s, *J* = 5.2 Hz, 2H), 2.68 (t, *J* = 5.8 Hz, 2H), 2.61 (t, *J* = 5.7 Hz, 2H), 1.98–1.94 (m, 2H), 1.92–1.89 (m, 2H), 1.67 (s, 1H), 1.44 (s, 1H). ^13^C NMR (126 MHz, CDCl_3_) δ 175.34, 165.75, 160.43, 158.23, 156.26, 143.13, 139.75, 139.58, 133.34, 131.01, 128.34, 122.67, 122.28, 116.81, 116.24, 116.06, 108.13, 103.37, 94.64, 79.64, 47.71, 38.42, 28.75, 28.51, 28.04, 27.34, 24.51, 21.37. MS: calcd. for C_33_H_38_BrN_2_O_4_^+^ [M^+^]: 605.2009; HRMS found: *m*/*z* 605.2011.

#### 4.4.2 Synthesis of Hemi-Br

Compound 5 (0.11 g, 0.18 mmol) was subjected to Boc deprotection in the presence of TFA/DCM (3/3 mL) at room temperature. After completing the reaction, the solvent was evaporated under a vacuum, cold Et_2_O was added to the crude solid, and the dark blue precipitate was collected by filtration to afford Hemi-Br (0.086 g, 94% yield). ^1^H NMR (500 MHz, DMSO) δ 8.57 (d, *J* = 14.8 Hz, 1H), 7.87 (*br.* s, 3H), 7.77 (d, *J* = 7.4 Hz, 1H), 7.69 (d, *J* = 8.0 Hz, 1H), 7.55 (t, *J* = 7.7 Hz, 1H), 7.46 (dd, *J* = 13.4, 5.6 Hz, 2H), 7.07 (s, 1H), 6.53 (d, *J* = 14.8 Hz, 1H), 4.46 (t, *J* = 7.2 Hz, 2H), 2.99 (m, 2H), 2.73 (t, *J* = 5.8 Hz, 2H), 2.68 (t, *J* = 5.8 Hz, 2H), 2.10–2.00 (m, 2H), 1.86–1.80 (m, 2H), 1.76 (s, 6H). ^13^C NMR (126 MHz, DMSO) δ 177.37, 160.53, 156.22, 153.01, 145.10, 141.95, 141.36, 140.68, 132.87, 131.27, 128.89, 127.01, 126.81, 122.89, 115.61, 114.32, 112.91, 104.07, 102.79, 50.37, 42.05, 36.39, 28.39, 27.52, 25.54, 23.59, 19.92. MS: calcd. for C_28_H_30_BrN_2_O_2_^+^ [M^+^]: 505.1485; HRMS found: *m*/*z* 505.1481.

### 4.5 Electrostatic Loading of Hemi-Br

Further, 1.0 mg/mL AS-GSH QDs (in water, pH = 9.5) were titrated with 50 μg/mL Hemi-Br (in water, pH = 9.0) using an isothermal titration calorimeter (Affinity ITC), and the enthalpy change resulting from the interactions was recorded. The flow rate was set to 15 μL/s and the results were reported as the change in the enthalpy (μcal) per injection.

### 4.6 Characterization

Absorbance spectra of NPs were recorded in the 300–1000 nm range using a Shimadzu UV–vis–NIR spectrophotometer. Photoluminescence spectra (PL) were recorded between 600 and 1100 nm (λ_ex_: 532 nm) using a custom-made PL instrument consisting of a DPSS laser (532 nm) and a 590 nm long-pass filter for the excitation and emission spectra, respectively. For the collection of the luminescence signal, a 1/8 Newport Cornerstone 130 monochromator and a femtowatt sensitive Si detector (Thorlabs PDF10A, 1.4 × 10^–15^ W Hz^–1/2^) were used. Hydrodynamic sizes and ζ-potentials were measured by a Malvern Zetasizer Nano ZS. The organic coating content of the QDs was determined by thermogravimetric analysis (TGA). Loading of free Hemi-Br to QDs was confirmed by isothermal titration calorimetry (Affinity ITC). The Ag content of prepared QDs was determined using an Agilent 7700X ICP-MS (inductively coupled plasma-mass spectrometer). Samples were digested with nitric acid and sulfuric acid (9:1 v/v). Crystal size and shapes of NPs were determined with a transmission electron microscope (TEM, Hitachi High-Tech Corporation). Fourier transform infrared spectroscopy (FTIR) was conducted with a Thermo Scientific Nicolet iS10 instrument in the wavenumber region from 680 to 4000 cm^–1^ with a 4 cm^–1^ resolution for functional group analysis.

### 4.7 Investigation of PDT/PTT Potential in Solution

#### 4.7.1 Solution Heating

The PTT potential of the solvent, QDs, and Hemi-Br in solution was investigated by irradiating 0.75 mL of aqueous solutions of QDs at 300 μg Ag/mL and 53 μg Hemi-Br/mL concentrations with a continuous wave (CW) fiber-coupled diode laser at 640 nm with an incident power of 215 mW (Thorlabs, L638P700M). Temperature increase (Δ*T*) was continuously monitored for 20 min with a thermocouple and a thermal camera simultaneously. Furthermore, the QDs were subjected to three cycles of laser on/off irradiation under the same conditions to investigate whether the photothermal effect is recoverable for the same solution upon consecutive irradiations. A concentration-dependent temperature increase of the irradiated AS-GSH-FA/Hemi-Br solutions was measured between 50 and 300 μg Ag/mL concentrations. Light-to-heat conversion efficiencies were calculated as reported previously.^56^ Briefly, the average steady-state temperature distribution on the cuvette surface was determined with a thermal camera. The parameters for temperature increase, incident laser power, and the mass (concentration) of the solution were arranged in such a way that the maximum temperature increase did not exceed 15 °C and a linearized heat transfer model was performed to analyze the data.

#### 4.7.2 Singlet Oxygen Generation

To investigate the singlet oxygen production potential in solution, the change in the emission of commercially available singlet oxygen sensor green (SOSG) dye was monitored using a free Hemi-Br solution at a 10 μM Hemi-Br concentration. Samples were prepared in PBS buffer (3 mL) containing 10% MeOH and 5 μM SOSG in MeOH was added. The laser irradiation of the solutions was performed at 640 nm (215 mW, 5 min) and the change in the green emission at 530 nm (ex: 504 nm) was measured using an Agilent spectrophotometer.

The singlet oxygen generation capacity of Hemi-Br was determined by using 1,4-diphenylbenzofuran (DPBF) in DMSO–PBS buffer (10 μM, pH 7.4, 99:1, %v/v). Methylene blue (Φ_Δ_ = 0.49 in DMSO) was used as a reference compound for singlet oxygen quantum yield calculation. In a typical procedure, Hemi-Br and DPBF were mixed in oxygen-bubbled DMSO and PBS. First, measurement was taken in dark, and the solution was exposed to light (640 nm) from a 10 cm distance and a 10 s time interval. The absorbance of DPBF was determined after each irradiation to evaluate ^1^O_2_ production. The slope of the absorbance maxima DPBF at 417 nm versus time graph was drawn. Finally, singlet oxygen quantum yield was calculated according to the equation given below
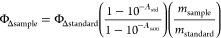
where the sample and the standard represent Hemi-Br and methylene blue, respectively, *m* is the slope of absorbance maxima of DPBF at 417 nm versus time graph, and *A* is the absorbance value of both the sample and the standard at an irradiation wavelength of 640 nm.

### 4.8 Cell Culture

Human cervical HeLa and human lung A549 cancer cell lines were grown in an RPMI 1640 medium, and healthy mouse fibroblast L929 cells were grown in a DMEM medium at 37 °C in a 5% CO_2_ humidified incubator. All culture media were supplemented with 10% fetal bovine serum and 1% penicillin–streptomycin.

### 4.9 Cytotoxicity Assay

The dose- and time-dependent cytotoxicities of Hemi-Br, AS-GSH, AS-GSH-FA, and AS-GSH-FA/Hemi-Br were investigated on HeLa, A549, and L929 cells using standard MTT assay. Cells were seeded at a density of 1 × 10^4^ cells per well into 96-well plates and incubated with free Hemi-Br and prepared NPs for 6 and 24 h between 0.5 and 12.5 μg Hemi-Br/mL or 2.85–71.25 μg Ag/mL concentrations. Then, the medium was removed, and cells were treated with 50 μL of an MTT (5 mg/mL in PBS) solution and 150 μL of a fresh medium for 4 h more. Then, DMSO/ethanol (1:1 v/v) was added to dissolve purple formazan crystals formed by the viable cells. The absorbance at 570 nm was recorded by a Synergy H1, Biotek Instruments microplate reader with a reference reading at 650 nm. Cells that were not treated with particles were used as controls. The relative cell viability was calculated using the following formula
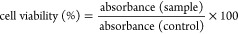


### 4.10 Cellular Uptake

Cells were seeded at a density of 175 000 cells/well in 3 mL glass-bottom Petri dishes and treated with AS-GSH and AS-GSH-FA at 50 μg Ag/mL for 6 h. The medium was removed, and cells were washed three times with PBS. Then, cells were incubated with 4% paraformaldehyde for 20 min, washed with PBS, and stained with DAPI (10 μg/mL). After 15 min of incubation, cells were rewashed three times with PBS to remove the unbound dye, and 1 mL PBS was left in each well to protect cells against drying. Cells that were not treated with the test materials were used as controls. Images were obtained by a confocal microscope (Leica dmi8/SP8) using filters for DAPI (λ_ex_ = 325–375 nm and λ_em_ = 435–485 nm) and NIR fluorescence (λ_ex_ = 510–550 nm and λ_em_ = 710 nm long pass).

Cellular uptake was quantitatively analyzed by measuring the intracellular NIR emission signal of cells after 6 h of incubation with the agents. Cells were seeded at a density of 17 500 cells/well in a 96-well plate and treated with AS-GSH, AS-GSH-FA, AS-GSH-FA/Hemi-Br, and free Hemi-Br at 57 μg Ag/mL or 10 μg Hemi-Br/mL concentrations for 6 h. The cells were washed with PBS to remove uninternalized particles, and the fluorescence intensity from each well was measured with a Synergy H1 (Biotek Instruments) microplate reader equipped with a NIR filter set (ex/em: 528/818 nm).

### 4.11 ROS Generation

HeLa and A549 cells were seeded and incubated with free Hemi-Br and AS-GSH-FA/Hemi-Br for 6 h as described above. Then, cells were treated with 10 μM DCFH-DA for 40 min and irradiated for 5 min with a 640 nm laser (300 mW, 0.78 W/cm^2^). Fluorescence intensity was read directly after laser irradiation using a microplate reader (Synergy H1, Biotek Instruments) at ex/em = 485/538 nm.

### 4.12 Singlet Oxygen Inhibition *In Vitro*

HeLa and A549 cells were seeded and treated with free Hemi-Br and AS-GSH-FA/Hemi-Br as mentioned in ROS generation experiments at 7.5 and 10 μg Hemi-Br/mL doses. After 6 h of incubation with the particles, the medium was replenished with a fresh medium containing 5 mM NaN_3_. Then, 5 min of laser irradiation at 640 nm (300 mW, 0.78 W/cm^2^) was applied, followed by overnight incubation. The cell viability was determined using MTT assay as described in previous protocols.

### 4.13 *In Vitro* Laser Irradiation

HeLa and A549 cells were seeded and treated with free Hemi-Br, AS-GSH, AS-GSH-FA, and AS-GSH-FA/Hemi-Br between 1.0 and 7.5 μg Hemi-Br/mL or 5.7 and 42.75 μg Ag/mL concentrations. After 6 h of incubation, the medium was replenished with a fresh medium, and cells were irradiated for 5 min with a 640 nm laser (300 mW, 0.78 W/cm^2^) from the bottom of the plate. Cell viability was determined 24 h after irradiation using an MTT assay. The same laser conditions were applied to cells that were not treated with free Hemi-Br or prepared NPs to investigate the laser power safety. Cells that were not treated with laser or test materials were used as controls.

### 4.14 Live/Dead Assay

Live/dead viability assay was used to observe the cell death before and after different treatments for both cell lines. HeLa and A549 cells were treated with AS-GSH, AS-GSH-FA, AS-GSH-FA/Hemi-Br, and free Hemi-Br for 6 h at 42.75 μg Ag/mL and 7.5 μg Hemi-Br/mL concentrations and irradiated with a 640 nm (300 mW, 0.78 W/cm^2^) laser for 5 min. Cells were then washed with PBS and stained with ethidium bromide (4 μM) and calcein-AM (2 μM) to image dead (red) and live (green) cells, respectively. Fluorescence images were collected with a fluorescence microscope (Zeiss Axio observer Z1).

### 4.15 Mitotracker Assay

Mitotracker Green (MTG) FM staining was performed according to the manufacturer’s protocol to confirm Hemi-Br localization in the mitochondria. HeLa cells were first incubated with 7.5 μg/mL Hemi-Br (42.75 μg/mL Ag) for 6 h in 96-well plates before staining. The dye concentration was set at 25 nM and incubated for 30 min. Colocalization images were captured using a Carl Zeiss fluorescence microscope.

### 4.16 LDH Assay

Cell membrane integrity was examined using a lactate dehydrogenase (LDH) Assay Kit (Abcam, Cat. ab65393) following the manufacturer’s protocol. HeLa cells were treated with AS-GSH, AS-GSH-FA, AS-GSH-FA/Hemi-Br, and free Hemi-Br as described in laser studies at a concentration of 7.5 μg/mL Hemi-Br (42.75 μg/mL Ag) and irradiated with a 640 nm laser (300 mW, 0.78 W/cm^2^) for 5 min. LDH levels were determined both with and without laser irradiation.

### 4.17 Statistical Analysis

The statistical significance was determined using two-way ANOVA with Tukey’s multiple comparison test on GraphPad Prism 9 software (GraphPad Software, Inc.). All data were expressed as mean ± standard deviation (SD), and *p* < 0.05 was considered statistically significant.

## Supporting Information Available

Additional characterizations: synthetic scheme of Hemi-Br (Scheme S1), FTIR spectrum of the QDs (Figure S1), ITC exotherms for Hemi-Br titration (Figure S2), TEM histograms (Figure S3), colloidal stability analysis with UV and PL spectra and DLS results (Figure S4 and Table S1), concentration-dependent solution PTT results (Figure S5), SOSG test to measure singlet oxygen generation in solution (Figure S6); *in vitro* results: intracellular uptake quantification via luminescence signal measurement (Figure S7), MTT results for cellular viability at the high doses (Figure S8), ROS quenching experiment results with NaN_3_ (Figure S9), live/dead cellular viability assay (Figure S10), and Mitotracker assay correlation plots (Figure S11), and HRMS and NMR spectrum data (PDF)
