# Supplementary material for: Image-Guided Enhanced PDT/PTT Combination Therapy Using Brominated Hemicyanine-Loaded Folate Receptor-Targeting Ag2S Quantum Dots
Source: Bioconjug Chem. 2023 Apr 20;34(5):880–92. doi: 10.1021/acs.bioconjchem.3c00096 (PMC10197082; doi:10.1021/acs.bioconjchem.3c00096)
Supplement: Supplementary file 1 — bc3c00096_si_001.pdf [file bc3c00096_si_001.pdf]

Image-Guided Enhanced PDT/PTT Combination Therapy Using Brominated Hemicyanine Loaded Folate Receptor Targeting Ag_2S Quantum Dots

Eda Celikbas[1], Ayca Saymaz[1], Hande Gunduz[1,2], Irem Koc[3], Ece Cakir[3], Alphan Sennaroglu[4,5], Safacan Kolemen[1,4], Havva Yagci Acar[1,4*] and Kubra Onbasli[6*]*

[1]Department of Chemistry, Koç University, Rumelifeneri Yolu, Sariyer, Istanbul 34450, Türkiye

[2]Nanofabrication and Nanocharacterization Centre for Scientific and Technological Advanced Research, Koç University, Istanbul, Türkiye

[3]Graduate School of Materials Science and Engineering, Koç University, Rumelifeneri Yolu, Istanbul 34450, Türkiye

[4]KUYTAM, Koç University Surface Science and Technology Center, Koc University, Rumelifeneri Yolu, Istanbul 34450, Türkiye

[5]Departments of Physics and Electrical-Electronic Engineering, Koç University, Rumelifeneri Yolu, 34450 Istanbul, Türkiye

[6]Department of Metallurgical and Materials Engineering, 34469 Istanbul Technical University, Istanbul, Türkiye

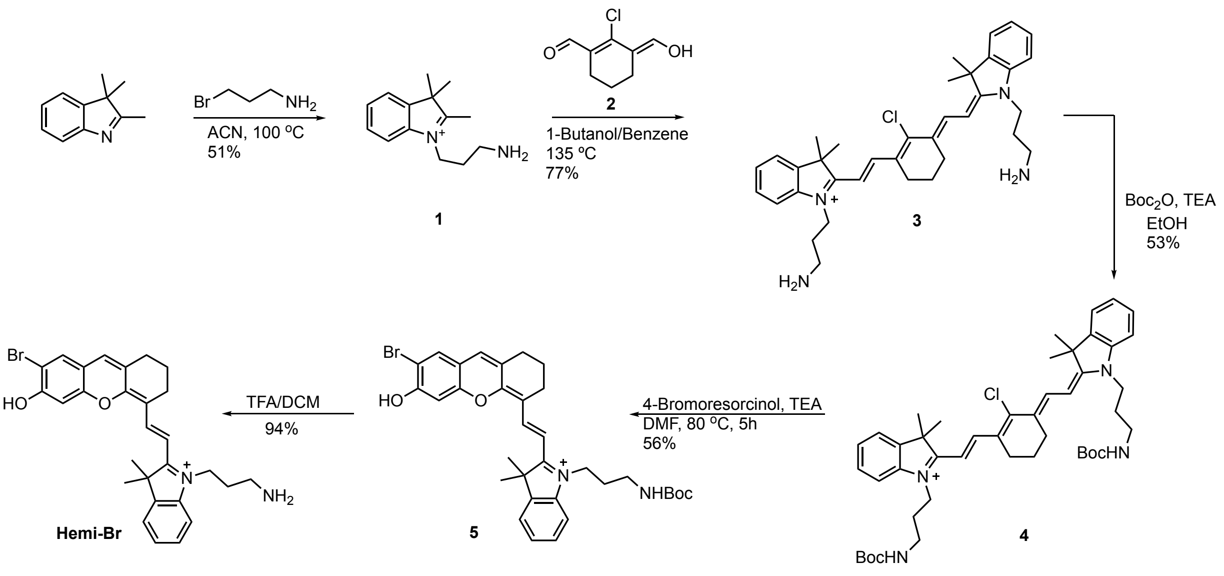

Scheme S 1 Synthetic pathway of Hemi-Br.

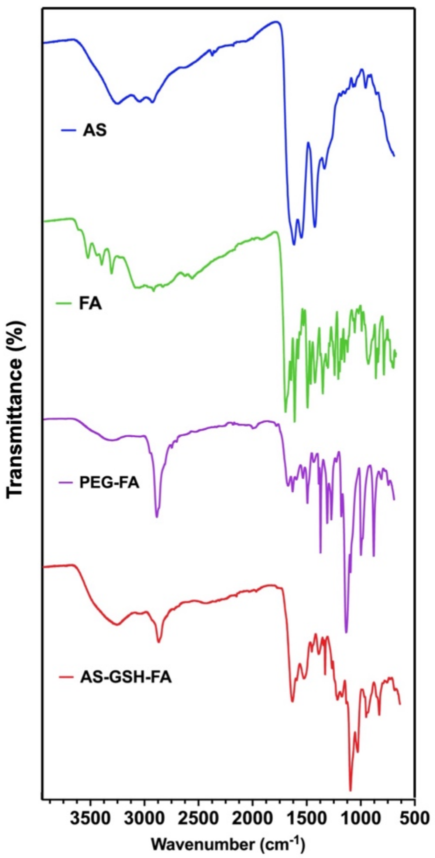

Figure S 1 FTIR spectrum of AS-GSH QDs, FA, PEG-FA and AS-GSH-FA QDs.

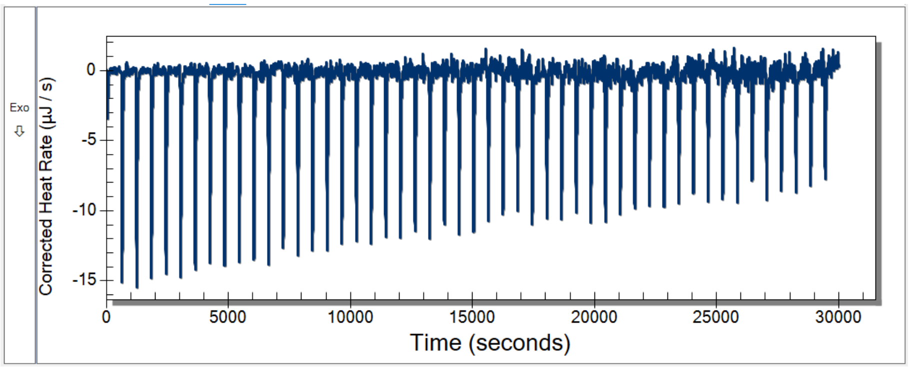

Figure S 2. ITC exotherm profile of the titration of AS-GSH-FA with Hemi-Br.

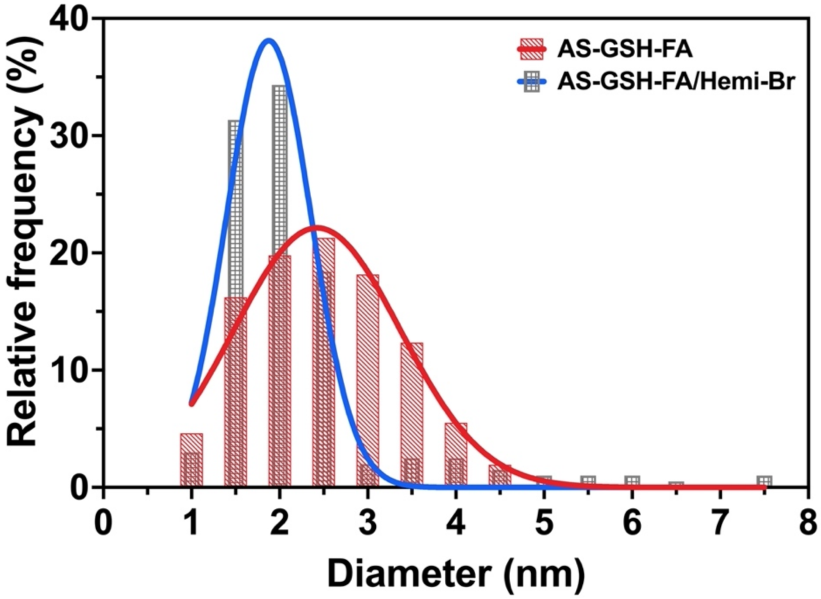

Figure S 3 TEM histograms of the QDs corresponding to Figure 2c-d.

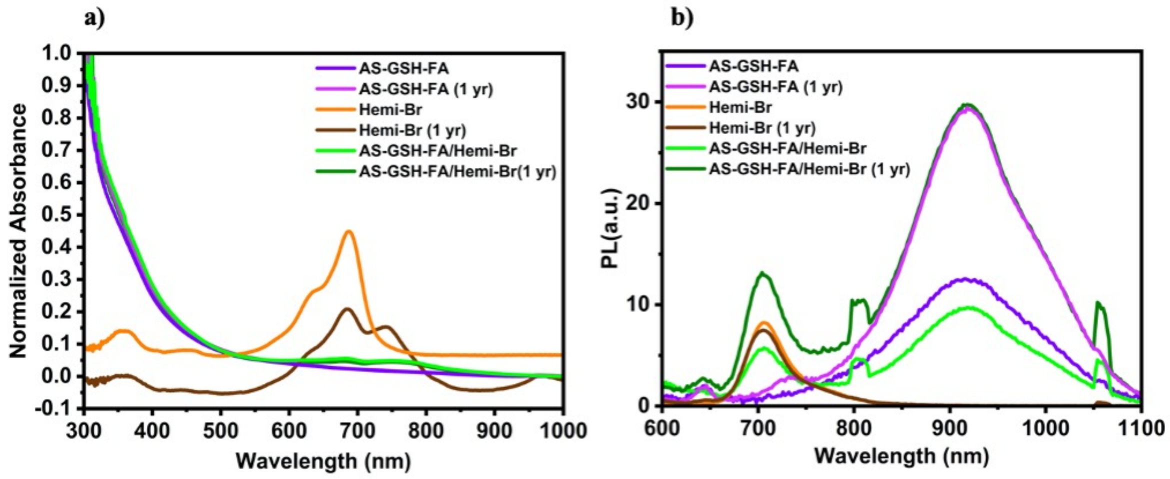

Figure S 4 Absorbance (a) and photoluminescence (b) spectra of the QD conjugates on day one and year one of the synthesis.

Table S 1 Hydrodynamic size and zeta potential of prepared QDs on day one and year one of the synthesis.

**Sample Name**	**PDI**	**Size by number (nm)**	**Size by intensity (nm)**	**Zeta Potential (mV)**
AS-GSH	0.17	2.94	7.21	-42.20
AS-GSH-FA	0.60	5.22	7.75	-34.70
AS-GSH-FA/Hemi-Br	0.47	75.50	112.20	-21.60
AS-GSH-FA (1 year)	0.16	5.07	7.61	-14.90
AS-GSH-FA/Hemi-Br (1 year)	0.55	87.34	107.4	-16.70

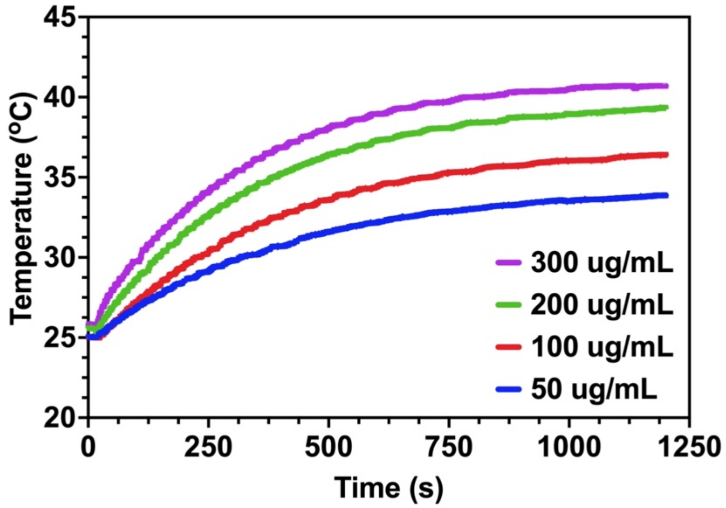

Figure S 5. Time and [Ag] concentration-dependent change in the temperature of AS-GSH-FA/Hemi-Br solutions irradiated at 640 nm, 215 mW.

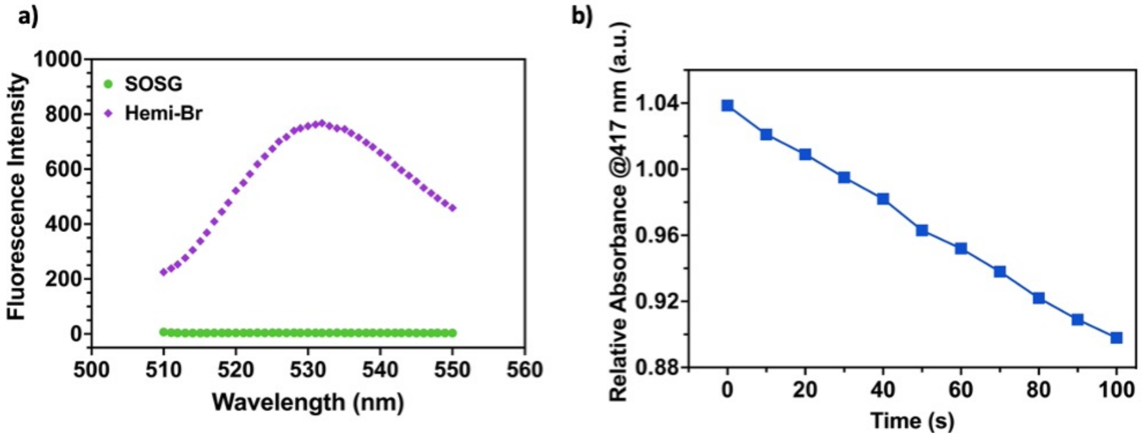

Figure S 6. (a) Fluorescence signal of SOSG at 525 nm upon irradiation at 640 nm (215 mW) of free Hemi-Br in 10% MeOH/PBS buffer (pH 7.4) at 7.5 μg Hemi-Br/mL as a spectrum. (b) Decrease in the absorption signal of DPBF with respect to time upon irradiation of Hemi-Br with a 640 nm light in DMSO:PBS (99:1, pH 7.4).

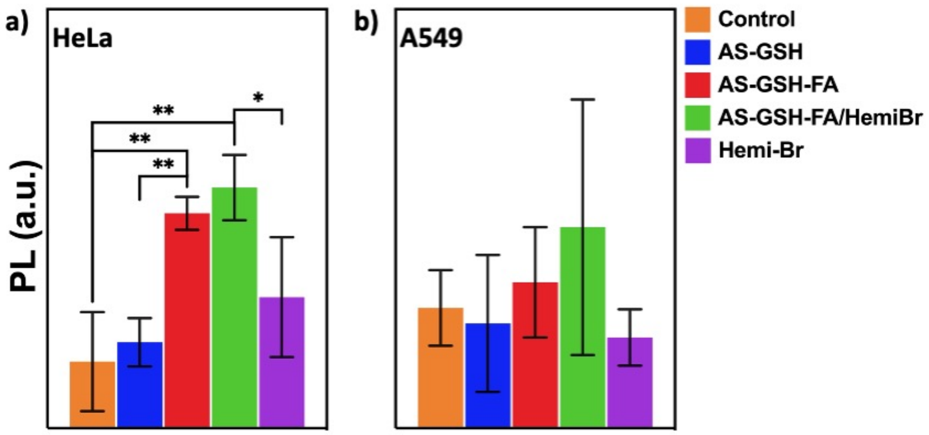

Figure S 7. Quantified intracellular NIR fluorescence signal measurement from (a) HeLa and (b) A549 cells treated with AS-GSH, AS-GSH-FA, AS-GSH-FA/Hemi-Br and free Hemi-Br for 6 h at 57 µg Ag/mL and 10 µg Hemi-Br/mL dose. The data are expressed as mean ± S.D. (n=3). (0.0332 (*), 0.0021 (**), 0.002 (***), < 0001 (****))

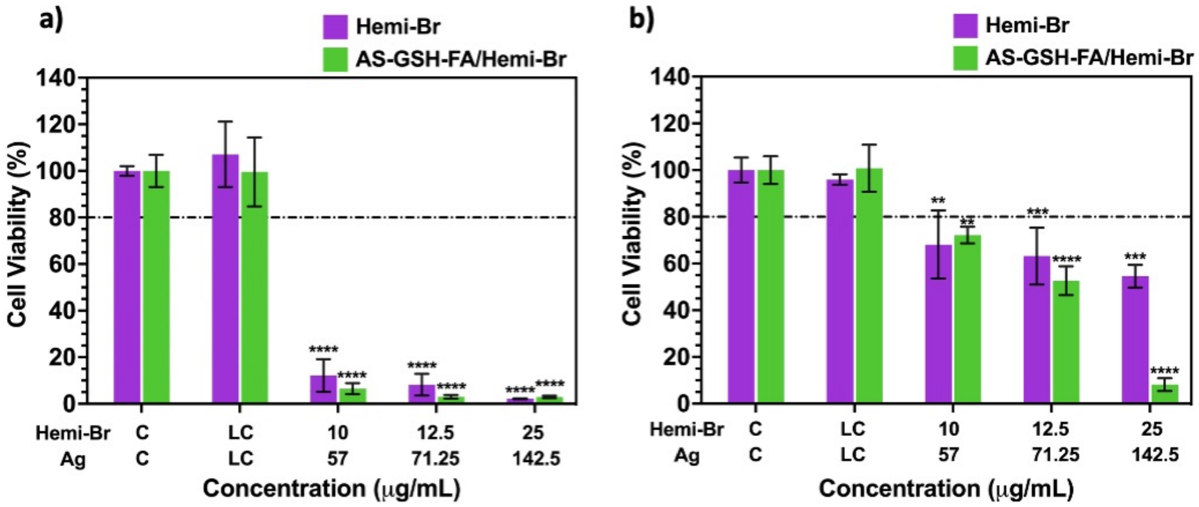

Figure S 8. Dose-dependent viability of (a) HeLa and (b) A549 cells treated with free Hemi-Br and QD-conjugates for 6 h and irradiated at 640 nm for 5 min at high doses. The data are expressed as mean ± S.D. (n=3). (0.0332 (*), 0.0021 (**), 0.002 (***), < 0001 (****))

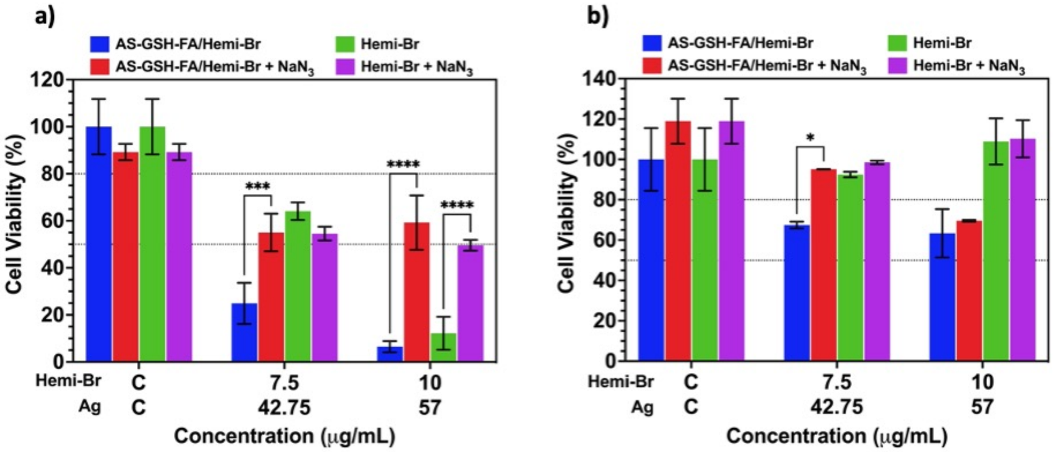

Figure S 9. Cell viabilities of (a) HeLa and (b) A549 cells treated with Hemi-Br and AS-GSH-FA/Hemi-Br upon laser irradiation (640 nm, 300 mW) for 5 min in the absence or presence of NaN_3 (5 mM). (0.0332 (*), 0.0021 (**), 0.002 (***), < 0001 (****))

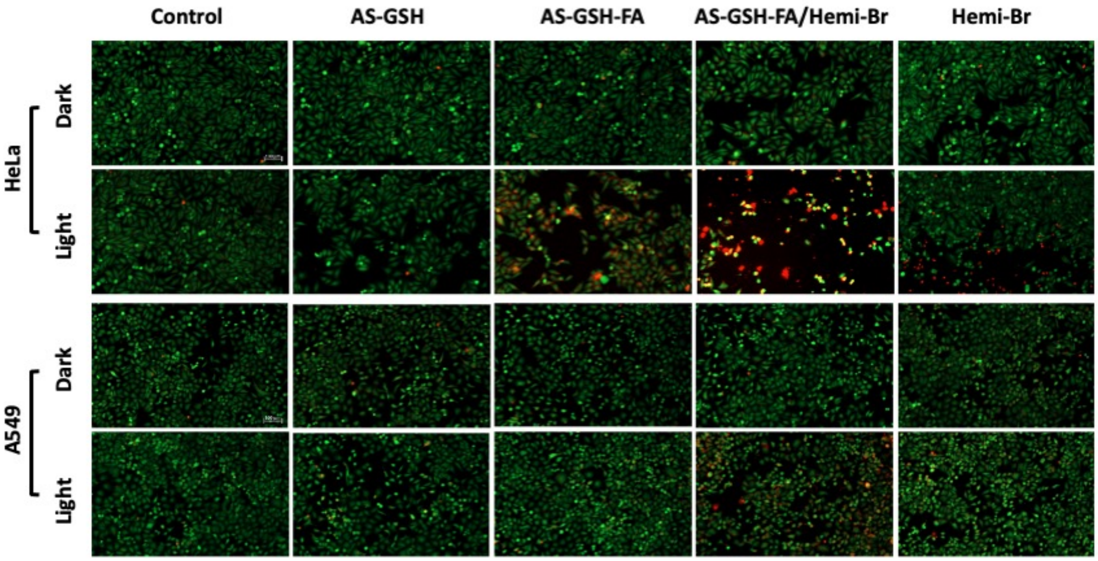

Figure S 10. Fluorescence microscopy images of HeLa and A549 cells treated with QDs at 42.75 μg Ag/mL and 7.5 μg Hemi-Br/mL concentrations for 6 h (Dark) followed by laser irradiation (640 nm, 300 mW, 5 min) (Light) after live/dead staining. "Control" indicates untreated cells.

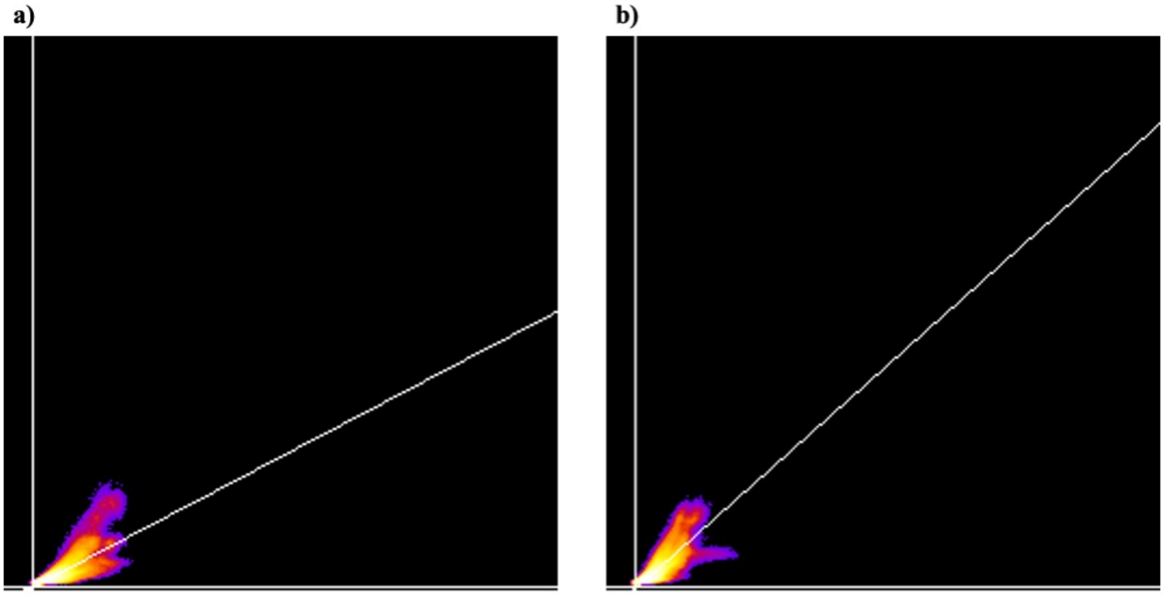

Figure S 11. Pearson correlation plots of MTG and (a) free Hemi-Br and (b) AS-GSH-FA/Hemi-Br. Images were processed and analyzed with ImageJ software.

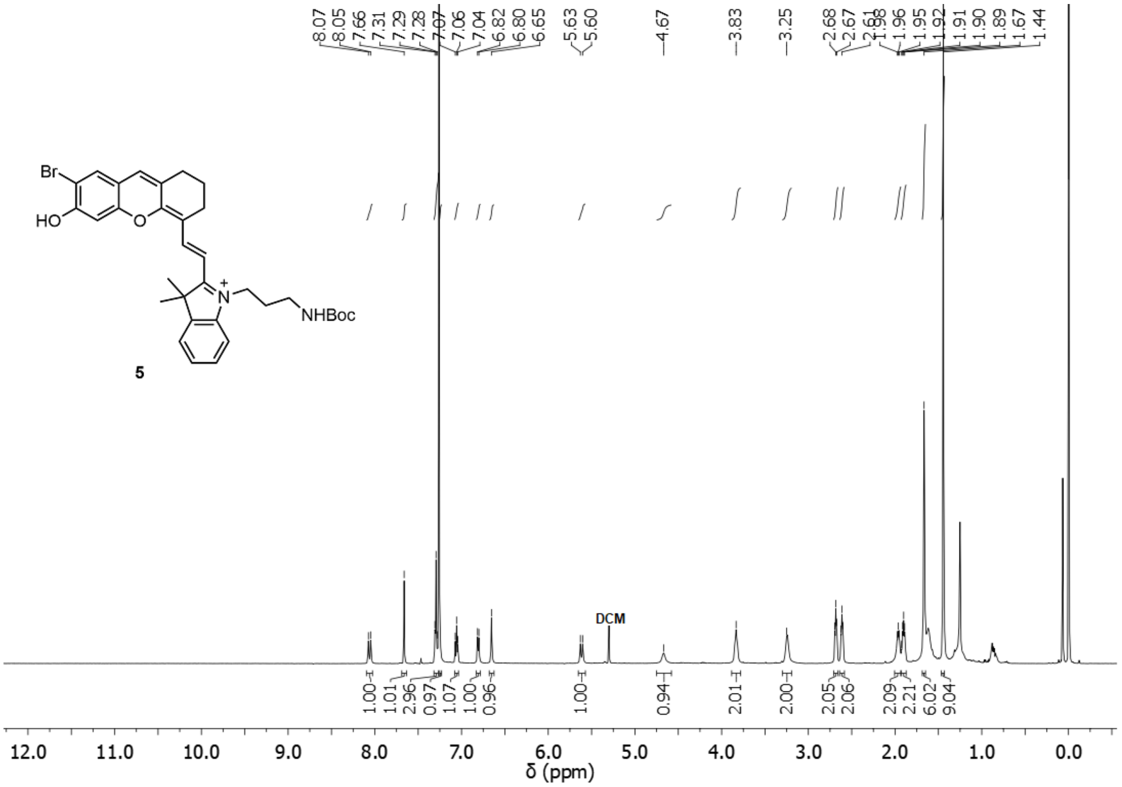

Figure S 9. ^{1}H NMR spectrum of compound **5** in $CDCl_3$.

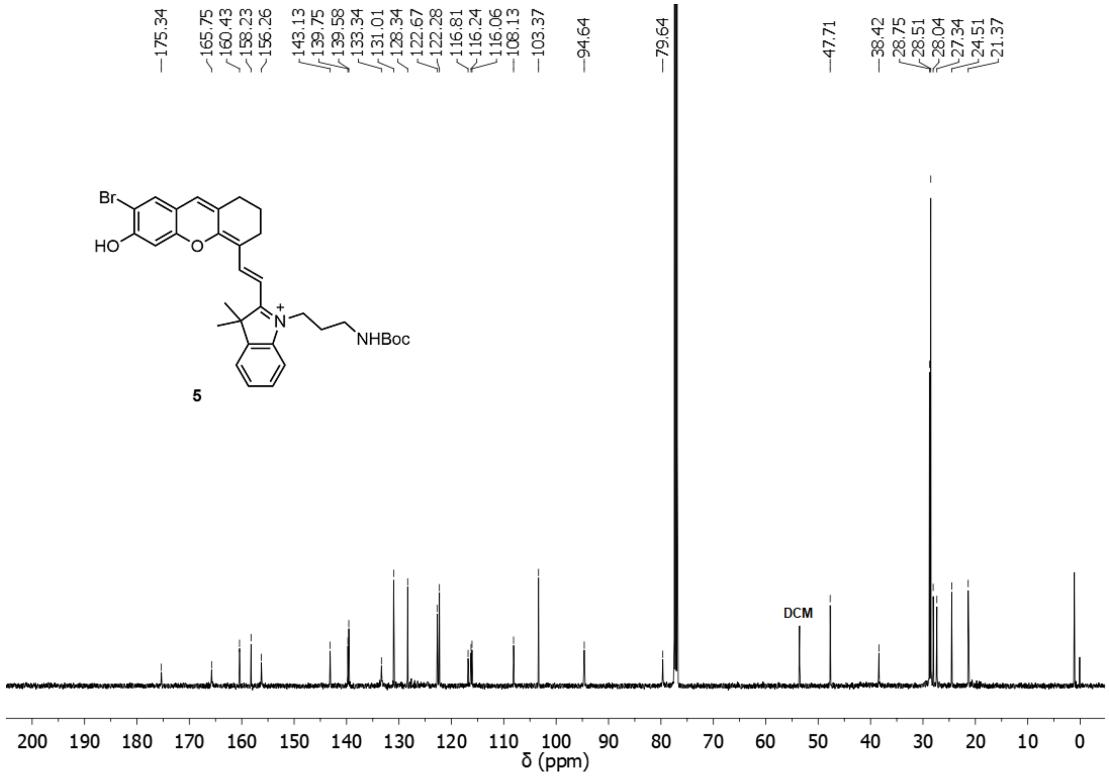

Figure S 10. ^{13}C NMR spectrum of compound **5** in $CDCl_3$.

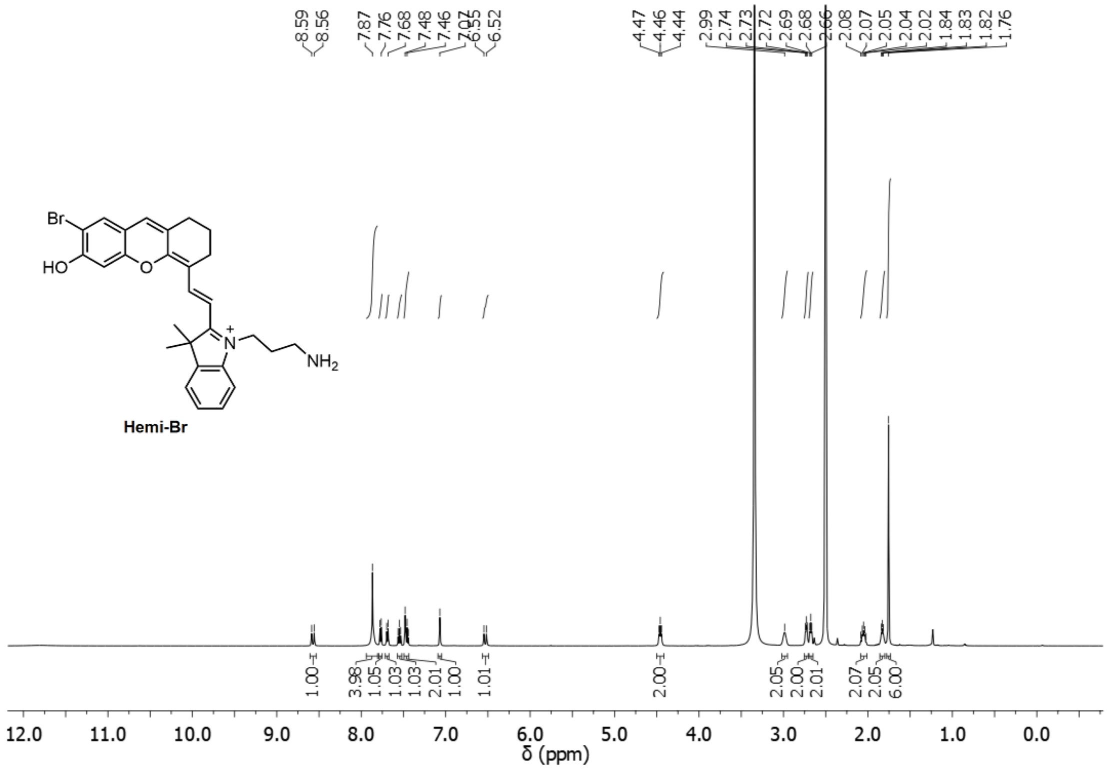

Figure S 11. ^{1}H NMR spectrum of compound **Hemi-Br** in DMSO-d_6.

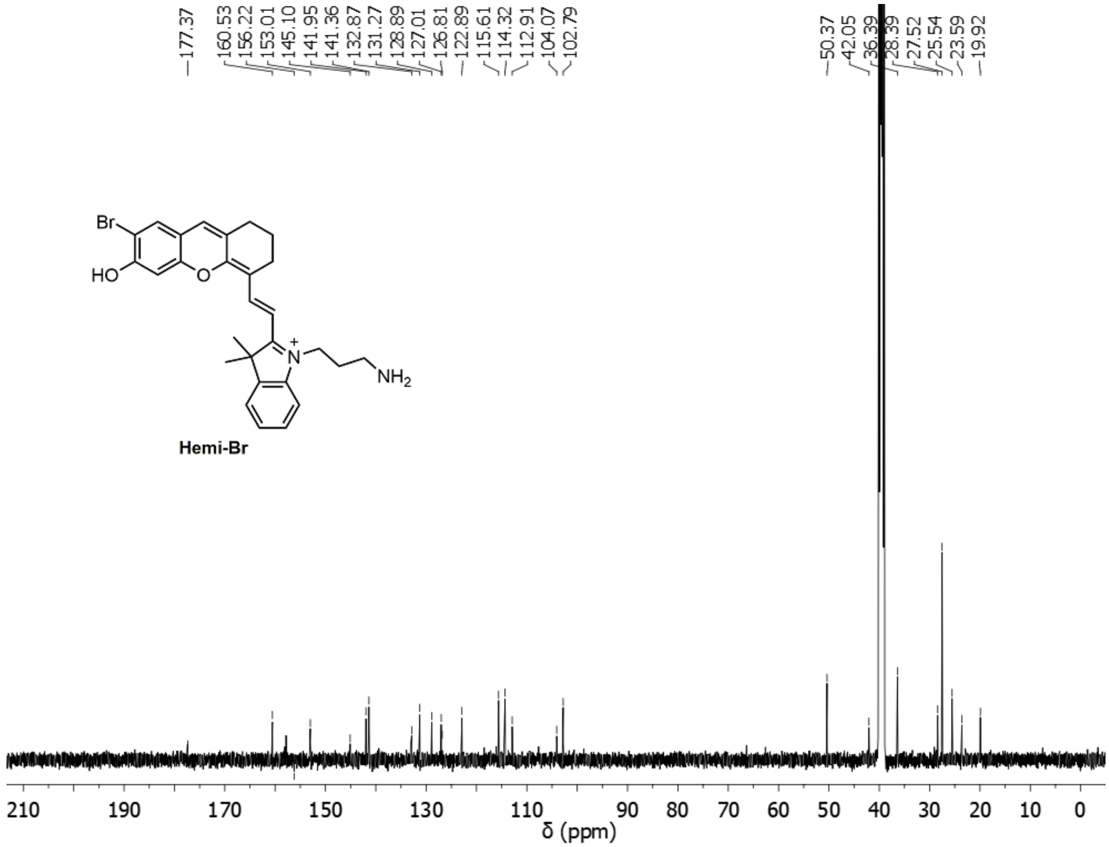

Figure S 12. ^{13}C NMR spectrum of compound **Hemi-Br** in DMSO-d_6

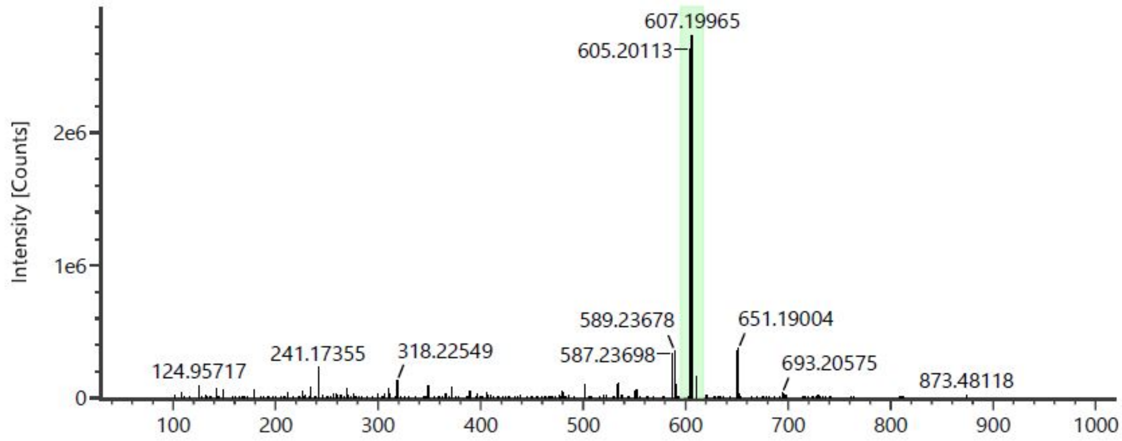

Figure S 13. HRMS spectrum of compound **5.**

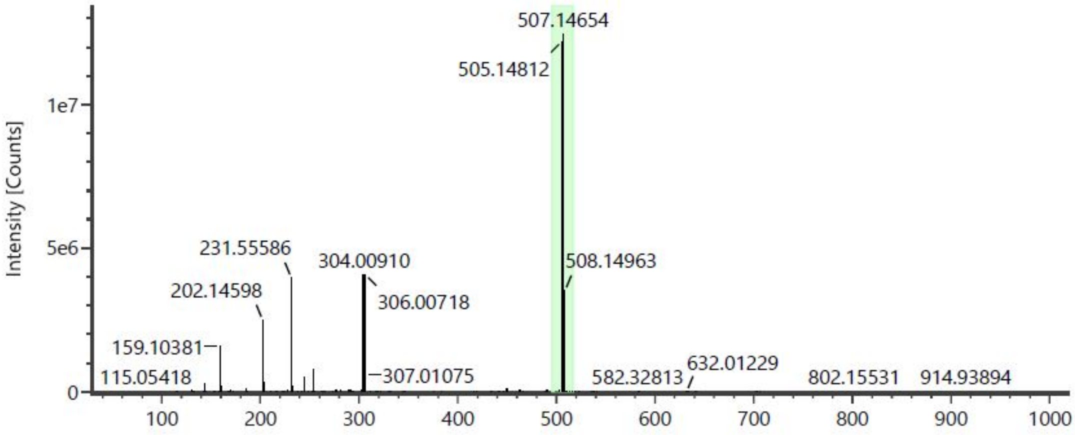

Figure S 14. HRMS spectrum of compound **Hemi-Br**.
